# PRMT6 facilitates EZH2 protein stability by inhibiting TRAF6-mediated ubiquitination degradation to promote glioblastoma cell invasion and migration

**DOI:** 10.1038/s41419-024-06920-2

**Published:** 2024-07-23

**Authors:** Ji Wang, Shiquan Shen, Jian You, Zhaotao Wang, Yan Li, Yanming Chen, Yonghua Tuo, Danmin Chen, Haoming Yu, Jingbo Zhang, Fangran Wang, Xiao Pang, Zongyu Xiao, Qing Lan, Yezhong Wang

**Affiliations:** 1 Department of Neurosurgery, Institute of Neuroscience, The Second Affiliated Hospital, Guangzhou Medical University, 510260 Guangzhou, China; 2 Department of Neurosurgery, The Affiliated Hospital, Southwest Medical University, 646000 Luzhou, China; 3 Department of Cardiology, The First Affiliated Hospital of University of Science and Technology of China, 230001 Hefei, China; 4 Department of Neurosurgery, The Second Affiliated Hospital of Soochow University, 215004 Suzhou, China; 5 Department of Neurosurgery, The Fourth Affiliated Hospital of Soochow University, 215124 Suzhou, China

**Keywords:** CNS cancer, Epigenetics, Genetics research

## Abstract

Invasion and migration are the key hallmarks of cancer, and aggressive growth is a major factor contributing to treatment failure and poor prognosis in glioblastoma. Protein arginine methyltransferase 6 (PRMT6), as an epigenetic regulator, has been confirmed to promote the malignant proliferation of glioblastoma cells in previous studies. However, the effects of PRMT6 on glioblastoma cell invasion and migration and its underlying mechanisms remain elusive. Here, we report that PRMT6 functions as a driver element for tumor cell invasion and migration in glioblastoma. Bioinformatics analysis and glioma sample detection results demonstrated that PRMT6 is highly expressed in mesenchymal subtype or invasive gliomas, and is significantly negatively correlated with their prognosis. Inhibition of PRMT6 (using *PRMT6* shRNA or inhibitor EPZ020411) reduces glioblastoma cell invasion and migration in vitro, whereas overexpression of PRMT6 produces opposite effects. Then, we identified that PRMT6 maintains the protein stability of EZH2 by inhibiting the degradation of EZH2 protein, thereby mediating the invasion and migration of glioblastoma cells. Further mechanistic investigations found that PRMT6 inhibits the transcription of *TRAF6* by activating the histone methylation mark (H3R2me2a), and reducing the interaction between TRAF6 and EZH2 to enhance the protein stability of EZH2 in glioblastoma cells. Xenograft tumor assay and HE staining results showed that the expression of PRMT6 could promote the invasion of glioblastoma cells in vivo, the immunohistochemical staining results of mouse brain tissue tumor sections also confirmed the regulatory relationship between PRMT6, TRAF6, and EZH2. Our findings illustrate that PRMT6 suppresses *TRAF6* transcription via H3R2me2a to enhance the protein stability of EZH2 to facilitate glioblastoma cell invasion and migration. Blocking the PRMT6-TRAF6-EZH2 axis is a promising strategy for inhibiting glioblastoma cell invasion and migration.

## Introduction

Glioblastoma, WHO grade IV glioma, is known for its aggressive characteristic and rapid progression, making it the most prevalent malignant brain tumor, responsible for 50.1% of all central nervous system malignancies [1, 2]. Despite notable advancements in diagnosis and treatment, the median survival period for glioblastoma patients remains a mere 14.6 months [3]. The challenge of complete surgical removal of glioblastoma is largely attributed to the infiltration of surrounding brain tissue by glioblastoma cells, resulting in high recurrence rates [4]. Extensive research conducted over several decades has revealed multiple mechanisms linked to the invasive characteristics of glioblastoma [5]. Summarizing the available studies, these mechanisms range from genetic to protein levels, involving aberrant invasive molecules and their corresponding signaling pathways [5–8]. Multiple epigenetic factors, such as aberrant DNA or RNA methylation and altered protein modification status, play a role in the invasion and migration of glioblastoma cells by modifying related molecules [9, 10]. Recent studies on glioblastoma have demonstrated that ubiquitination [11], acetylation [12], and methylation [13], which are common forms of post-translational modifications, contribute to promoting glioblastoma invasion.

Protein arginine methyltransferases (PRMTs) are enzymes that are essential for the post-translational modification of proteins through the addition of methyl groups to arginine residues. These enzymes have been linked to tumor invasion by controlling gene transcription through histone methylation or directly methylating associated proteins [14]. Recent studies have revealed significant findings regarding the involvement of PRMT3 in augmenting HIF1A-induced glycolysis and metabolic reprogramming, thereby promoting glioblastoma advancement [15]. Additionally, elevated levels of PRMT5 have been associated with enhanced invasion of glioblastoma cells [16]. PRMT6, a type I PRMT within the PRMT family, stands out for its ability to catalyze the asymmetric dimethylation of histone 3 at arginine 2 (H3R2me2a), which plays a crucial role in the epigenetic control of gene transcription. Moreover, PRMT6 methylates a diverse array of cellular proteins to modulate their functions [17]. Notably, dysregulation of PRMT6 expression, which has been consistently found in numerous studies, correlates with the aggressiveness of various human cancers such as prostate cancer, lung cancer, and gastric cancer [18]. *PRMT6* has been identified as an oncogene that promotes cell mitosis and proliferation in glioblastoma [19, 20], highlighting its significance in glioblastoma and suggesting its potential as a therapeutic target. However, it remains to be determined whether PRMT6 is involved in the invasion process of glioblastoma and the underlying mechanism. Therefore, further research is necessary to gain a full understanding of the impact of PRMT6 on glioblastoma invasiveness and to provide more comprehensive evidence for targeted therapy in glioblastoma.

Enhancer of zeste homolog 2 (EZH2), a catalytic subunit of the polycomb repressive complex 2, possesses histone-lysine N-methyltransferase activity that catalyzes trimethylation of lysine 27 in histone 3 (H3K27me3), inducing chromatin compaction and preventing the transcription of target genes [21, 22]. The involvement of EZH2 in regulating gene transcription and its abnormal expression, which is linked to heightened proliferation and invasion of brain tumor cells, has attracted significant interest [23]. EZH2 serves as a crucial intermediary regulatory factor that enhances the proliferation, migration, and invasion of glioma cells [24–26]. Studies have demonstrated that reducing EZH2 levels can decrease the invasive, migratory, and proliferative abilities of glioma cells, while also promoting apoptotic processes [27]. EZH2 has been shown to be regulated by PRMT1 [28], CARM1 [29], and PRMT5 [30], while whether PRMT6 is a regulator of EZH2 in glioblastoma is the subject of further investigation.

In this study, we present findings that highlight the significant role of PRMT6 in the invasion of glioblastoma cells both in vitro and in vivo. Our mechanistic investigation reveals that PRMT6 exerts its influence by epigenetically suppressing the transcription of the *TRAF6* gene through H3R2me2a, consequently diminishing the ubiquitination and degradation of EZH2. Subsequent analysis demonstrates that the invasion of glioblastoma cells in vitro was effectively hampered by a specific PRMT6 small molecule-targeted inhibitor (EPZ020411) [19, 20]. These results strongly suggest that targeting the PRMT6-TRAF6-EZH2 axis could hold substantial promise as a prognostic indicator and therapeutic strategy for glioblastoma.

## Materials and methods

### Clinical specimens

This study utilized 40 human glioma tissues, comprising 11 samples of non-invasive low-grade glioma, 13 samples of invasive low-grade glioma, and 16 samples of glioblastoma. These tissues were obtained from patients who had been initially diagnosed with malignant gliomas at the Second Affiliated Hospital of Guangzhou Medical University in Guangzhou, Guangdong, China. All participants in this study received written informed consent.

### Cell culture

Human glioblastoma cell lines (U87, LN229), and HEK293T cells were sourced from the Culture Collection of the Chinese Academy of Sciences (Shanghai, China). All of the cells were cultured in DMEM (Gibco, MD, USA) supplemented with 10% fetal bovine serum (FBS; Gibco) and maintained at 37 °C in a 5% CO_2_ atmosphere.

### Plasmids, shRNAs, and siRNAs

The pcDNA3.1-*PRMT6*-FLAG, pcDNA3.1-*TRAF6*-HA, pcDNA3.1-*TRAF6*(C70A)-HA, pcDNA3.1-*EZH2*-FLAG, and pcDNA3.1-*Ubiquitin*-His plasmids were created by YouBio Biotechnology (Changsha, China). hU6MCS-Ubiquitin-shPRMT6 lentiviral shRNA plasmid and *TRAF6* siRNA were generated by GenePharma (Suzhou, China). The specific sequences for shPRMT6 and siTRAF6 are listed in Supplementary Table S1. Transfection of the plasmids or siRNA into cells was carried out with Lipofectamine 3000 reagent (Invitrogen, USA) according to the manufacturer’s instructions.

### Transwell assay

For the invasion experiment, Matrigel (DMEM 1:8 dilution, Corning, USA) was pre-coated in a transwell chamber (Corning, USA) and incubated at 37 °C for 2 h prior to the experiment. The upper chamber was seeded with cells (5 × 10^4^ cells in serum-free DMEM), while the lower chamber contained DMEM with 10% FBS. Following a 24 h incubation period, the Matrigel and cells in the upper chamber were removed using a cotton swab. Subsequently, the chambers were fixed with 4% paraformaldehyde (PFA) and stained with 0.5% crystal violet. Cells invading were imaged using an optical microscope and analyzed by ImageJ software.

### Wound healing assay

The wound healing assay is a commonly used method to test the migration of cells in vitro. Cells are seeded in a six-well plate and are left to reach 90–100% confluence by 24 h. To create a linear scratch on the cell surface, a 1.0 mL pipette tip is gently moved across the specified region. Detached cells and debris are removed by rinsing the dishes with sterile PBS, and images of the scratches are taken at the beginning of the experiment (0 h) using a microscope. The cells are then incubated in FBS-free DMEM for a period of time, typically 24 or 48 h, and images of the scratches are taken at the end of the incubation period. Cells migrating were imaged using an optical microscope and analyzed by ImageJ software.

### Protein co-immunoprecipitation (Co-IP) assay

The protein co-immunoprecipitation assay is a technique utilized to investigate protein-protein interactions. For the Co-IP assay, the protein was extracted from the cells using NP-40 lysis buffer. In the endogenous Co-IP assay, anti-EZH2 antibody (CST, #14866) or anti-TRAF6 antibody (CST, #3686), and control IgG as a negative control, were added to the cell lysate and incubated overnight at 4 °C. Subsequently, Protein A/G beads were introduced to the lysate-antibody mixture, binding to the antibody and allowing the entire protein complex to be precipitated from the lysate. The eluted proteins were then subjected to analysis using western blotting with anti-EZH2 and anti-TRAF6 antibodies to identify the interacting proteins. In the exogenous Co-IP assay, the anti-FLAG antibody and anti-HA were immobilized onto Protein A/G agarose beads (bimake, B26101/B26201, USA) and incubated separately overnight with the cell extracts at 4 °C. Finally, the beads were analyzed using western blotting with anti-HA, anti-FLAG, and anti-EZH2 antibodies.

### Chromatin immunoprecipitation (ChIP) qPCR

The cells were crosslinked with 1% PFA for 10 min at RT, followed by the addition of 0.125 M glycine to halt the crosslinking process. Subsequently, the cells were washed with pre-cold PBS, centrifuged, lysed using a buffer containing Protease Inhibitor Cocktail, and centrifuged again to isolate the cell nuclear. The cell nuclear was ultra-sonicated for 6 min with 4 s of ultra-sonication at 8-s intervals. It was then added elution buffer containing RNase A, and incubated with Proteinase K at 62 °C for 2 h. The fragmented chromatin extract was incubated with antibodies (PRMT6, H3R2me2a, IgG) overnight, followed by incubation with Protein A/G magnetic beads at 4 °C for 2 h. Following a comprehensive process of washing, elution, and reverse cross-linking, the DNA undergoes purification to prepare for qPCR analysis. Gel electrophoresis was conducted to examine the production of qPCR reaction. The primers utilized for ChIP-qPCR analysis within the promoter region can be found in Supplementary Table S2.

### RNA sequencing and tandem mass tag (TMT) analysis

Total RNA and total protein from U87 cells with PRMT6 knockdown or control were subjected to RNA sequencing and TMT analysis, as described previously [19]. The raw data of RNA sequencing was uploaded to GEO and the accession number is GSE221971.

### Xenograft tumor assay and Hematoxylin–Eosin (H–E) staining

Intracerebral xenograft tumor mouse model and HE staining were used to observe PRMT6-mediated glioblastoma cell invasion in brain tissue, as described previously [19].

### Statistical analysis

SPSS 21.0 (Chicago, USA) and GraphPad Prism 8.0 software were used for statistical analysis of the data. Bars and error represent the mean ± standard deviation (mean ± SD) of at least three independent replicate measurements. Unpaired Student *t*-tests were utilized to analyze the means of normally distributed continuous data between two groups. Survival curves were plotted by Kaplan–Meier and compared by log-rank test. Statistical significance was defined as *P* < 0.05.

Additional detailed methodology is available in Supplementary Materials.

## Results

### The expression of PRMT6 is positively correlated with the invasion of glioma

**Fig. 1:**
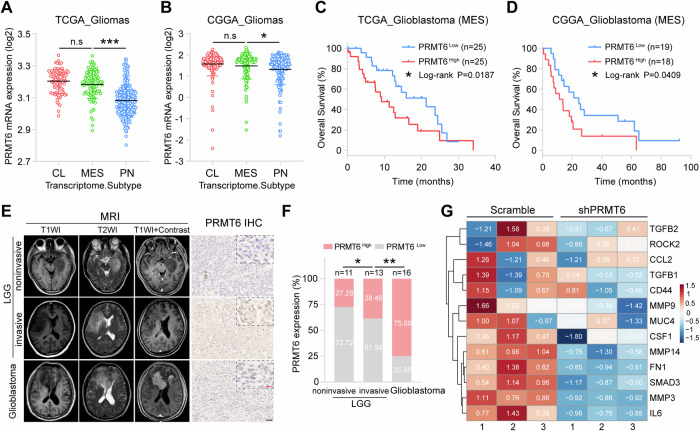
PRMT6 is positively associated with the invasiveness of glioblastoma. **A**, **B** The expression of PRMT6 in proneural (PN), classical (CL), and mesenchymal (MES) subtypes of glioblastoma patients from the TCGA (**A**) and CGGA (**B**) databases. **C**, **D** Kaplan–Meier curves showed the overall survival of patients according to *PRMT6* levels in patients with MES-subtype glioblastoma in the TCGA (**C**) and CGGA (**D**) databases. **E** Left: Representative MRI images of patients with noninvasive LGG, invasive LGG, and glioblastoma; Representative PRMT6 IHC images of patients with noninvasive LGG, invasive LGG, and glioblastoma. Bar: 10 μm (Red), 50 μm (Black). **F** The semi‑quantitative for the IHC results of PRMT6. **G** The RNA-seq heatmap displaying expression distribution of 13 genes associated with invasiveness in PRMT6 knockdown U87 cells and the control cells. n.s: no significant, *p < 0.05, **p < 0.01, ***p < 0.001.

Based on molecular signatures of patients’ tumors, glioma can be categorized into three distinct subtypes: Proneural (PN), Classical (CL), and Mesenchymal (MES), of which the prognosis of patients with CL and MES subtypes are relatively poor [31]. The heightened invasion and poorer prognosis observed in the MES subtypes can be attributed to the specific gene expression patterns within this subtype [32]. To investigate the potential correlation between PRMT6 expression in glioma tissues and their invasive characteristics, we analyzed *PRMT6* expression levels in three glioma subtypes patients from the CGGA and TCGA databases. The results showed that the expression of *PRMT6* in glioma of CL and ME subtypes was the highest, significantly higher than that in glioma of PN subtypes, and there was no significant difference in the expression of *PRMT6* in glioma of CL and ME subtypes (Fig. 1A, B). It suggests that the expression of PRMT6 in glioma may be related to cell proliferation and invasion. The prognostic significance of *PRMT6* expression in patients with MES-subtype glioblastoma was then analyzed, and the results revealed that patients with elevated *PRMT6* levels exhibited a poorer overall survival outcome in comparison to those with low *PRMT6* expression (Fig. 1C, D). Furthermore, we collected glioma specimens from 40 patients and divided them into PRMT6^Low^ (0–1) group and PRMT6^High^ (2–3) group according to IHC staining scores. In addition, low-grade gliomas (LGG) are divided into invasive LGG and noninvasive LGG based on MRI images. Analysis of IHC results of 40 samples found that the expression of PRMT6 in glioblastoma was significantly higher than that in LGG, and the expression of PRMT6 in invasive LGG was higher than that in non-invasive LGG (Fig. 1E, F). IHC results indicate that the expression of PRMT6 may be related to the aggressiveness of glioma. Subsequently, we further analyzed the previous transcriptome sequencing data and found that silencing PRMT6 in glioma cells can reduce the expression of invasion-related molecules (TGFB1/2, MMP3/9/14, FN1, ROCK2, etc.) (Fig. 1G). Taken together, these findings suggest that the expression of PRMT6 was highly positively correlated with invasion in glioma.

### PRMT6 induces the invasion and migration of glioblastoma cells

**Fig. 2:**
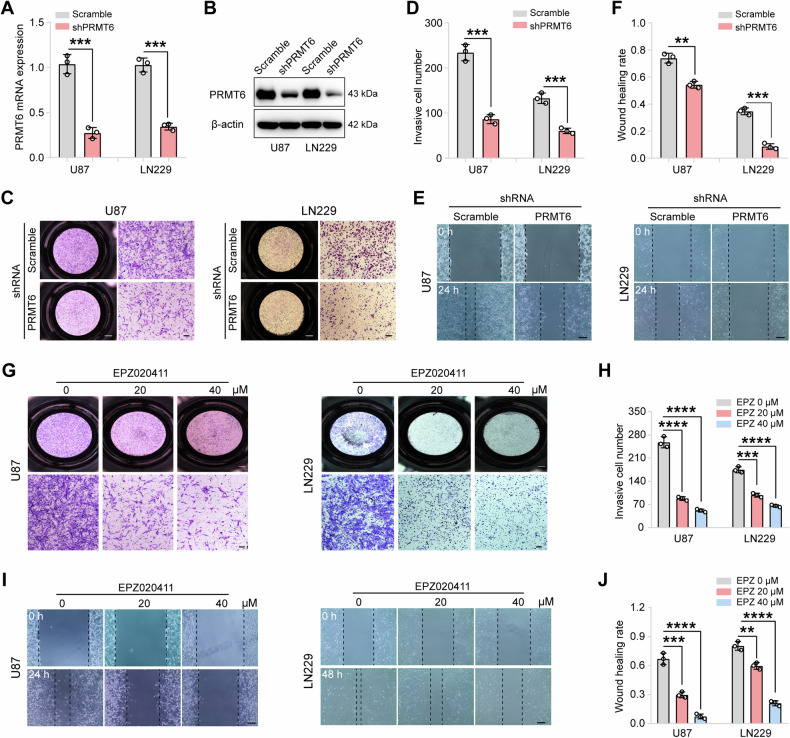
PRMT6 promotes the invasion and migration of glioblastoma cells. **A** qRT-PCR analysis was utilized to detect the mRNA expression levels of *PRMT6* in U87 and LN229 cells following transfection with either *PRMT6* shRNA or Scramble lentivirus. **B** The protein levels of PRMT6 were observed in U87 and LN229 cells that were transfected with either *PRMT6* shRNA or Scramble shRNA lentivirus by immunoblotting analysis. **C** Transwell assay was used to assess the invasion capacities in U87 and LN229 cells transfected with *PRMT6* shRNA or Scramble shRNA lentivirus. Bar: 100 μm. **D** Quantification of the invasive cells number. **E** The migration ability of PRMT6-knockdown U87 and LN229 cells or control cells was measured via a wound-healing assay. Bar: 200 μm. **F** Quantification of the wound healing rate. **G** Transwell assay analysis was utilized to assess the invasion abilities of U87 and LN229 cells when exposed to the designated EPZ020411 concentration. Bar: 100 μm. **H** Quantification of the number of invaded cells after treatment with the indicated concentrations of EPZ020411. **I** Wound-healing assay analysis to evaluate the migration ability of U87 and LN229 cells treated with specific concentrations of EPZ020411. Bar: 200 μm. **J** The wound healing rate was quantified after treatment with different concentrations of EPZ020411. **p < 0.01, ***p < 0.001, ****p < 0.0001.

To investigate the effect of PRMT6 expression on glioblastoma cell invasiveness, cell models were created with silenced or overexpressed PRMT6 through *PRMT6* shRNA lentivirus or *PRMT6* ORF plasmid transfection. The results from qRT-PCR and immunoblotting indicated a significant reduction in PRMT6 protein expression in LN229 and U87 cells (Fig. 2A, B), while an increase was observed in LN229 cells (Fig. S1A, B), which validates the successful construction of cell model. Subsequently, transwell assay was introduced and the results demonstrated that silencing PRMT6 notably decreased the invasion of LN229 and U87 cells (Fig. 2C, D), whereas overexpression of PRMT6 enhanced invasion in LN229 cells (Fig. S1C, D). Wound healing assay was also conducted to assess the migration effect of PRMT6 on glioblastoma cells. Examination of images depicting the degree of scratch healing revealed that the depletion of PRMT6 expression significantly suppressed the gap closure rate of glioblastoma cells (Fig. 2E, F), while PRMT6 overexpression strongly accelerated the gap closure of LN229 cells (Fig. S1E, F). In addition, to further verify the role of PRMT6 in glioblastoma invasiveness, we used PRMT6 inhibitors (EPZ020411) to treat glioblastoma cells for 48 h and repeated the above experiments and obtained similar results, indicating that inhibition of PRMT6 can weaken the invasiveness and migration of glioblastoma cells (Fig. 2G–J). Collectively, these findings indicate that elevated PRMT6 expression can the invasion and migration of glioblastoma cells.

### PRMT6 enhances the protein stability of EZH2 by attenuating its degradation

**Fig. 3:**
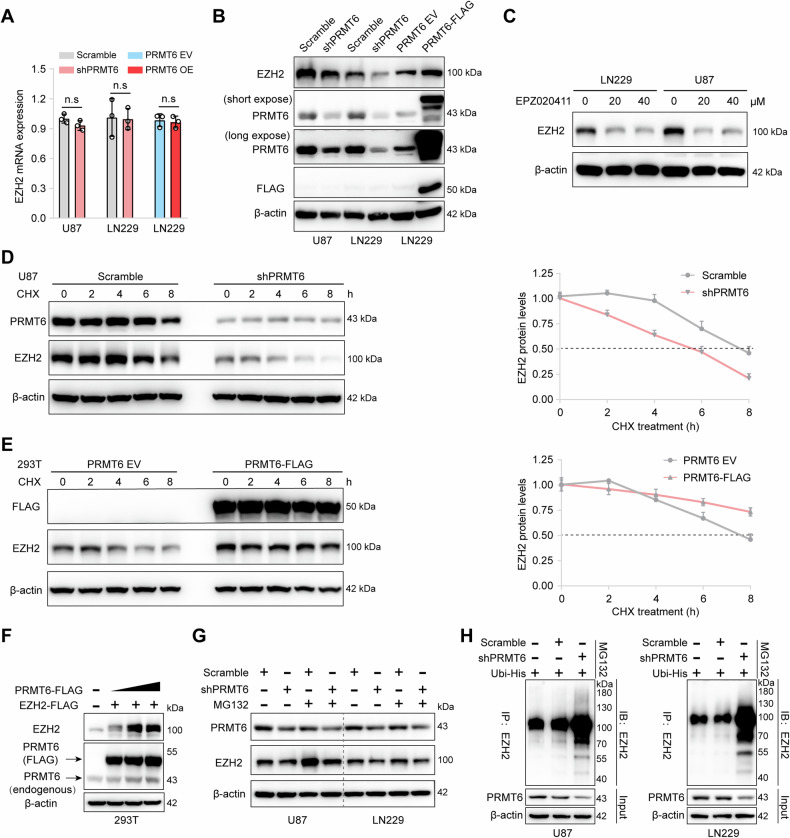
PRMT6 maintains the protein stability of EZH2 via the ubiquitin-proteasome pathway. **A** qRT-PCR analysis was used to determine the mRNA levels of *EZH2* in U87 and LN229 cells, both with and without PRMT6 knockdown, as well as in LN229 cells with or without PRMT6 overexpression. **B** The protein expression of EZH2 was detected by immunoblotting analysis in U87 and LN229 cells, both with and without PRMT6 depletion, as well as in LN229 cells with and without PRMT6 overexpression. **C** The expression of EZH2 protein in U87 and LN229 cells, upon treatment with varying concentrations of EPZ020411 for 48 h, was assessed via immunoblotting analysis. **D** The half-life of EZH2 protein in U87 cells with and without knockdown of PRMT6 was assessed and quantified by immunoblotting analysis. **E** Immunoblotting analysis was performed on HEK293T cells that were transfected with either PRMT6 overexpression or vector plasmids to measure and quantify the protein half-life of EZH2. **F** The protein expression levels of PRMT6 and EZH2 were assessed in HEK293T cells that were transfected with EZH2-FLAG and various amounts of PRMT6-FLAG plasmids by immunoblotting analysis. **G** U87 and LN229 cells, both with and without PRMT6 depletion, were exposed to either vehicle or MG132 (20 μM) for a duration of 6 h, and the abundance of EZH2 was examined by western blotting. **H** Cells U87 and LN229, with or without depletion of PRMT6, were transfected with His-Ubi and subsequently exposed to MG132 (20 μM) for a duration of 6 h. Following this treatment, cell lysates underwent immunoprecipitation using an anti-EZH2 antibody, enabling the detection of EZH2 ubiquitination via western blotting. n.s: no significant.

To elucidate how PRMT6 enhances the invasion of glioblastoma cells, we first analyzed the previously completed proteomic data [19] and found that the protein level of EZH2 was significantly down-regulated in PRMT6-deficient U87 cells. EZH2 has been confirmed to be a key regulator of enhanced glioma cell invasiveness [24]. Therefore, to explore whether PRMT6 has a regulatory effect on EZH2 in glioblastoma cells, we examined the influence of PRMT6 silencing or overexpression on EZH2 expression. qRT-PCR results showed that there was no significant difference in *EZH2* mRNA levels in PRMT6 knockdown or overexpression glioblastoma cells compared with control cells (Fig. 3A). Notably, western blotting analysis showed that EZH2 protein expression was decreased in PRMT6-silenced U87 and LN229 cells, while it was promoted in PRMT6-overexpressed LN229 cells (Fig. 3B). In addition, glioblastoma cells were treated with a PRMT6 inhibitor (EPZ020411) for 48 h, and it was found that EZH2 protein expression in glioblastoma cells was significantly inhibited (Fig. 3C), suggesting that PRMT6 may regulate the expression level of EZH2 through post-translational modification. Studies have confirmed that EZH2 can serve as a substrate in tumor cells and be ubiquitinated and degraded by the proteasome system, playing a role in inhibiting tumor progression [33]. To determine whether PRMT6 regulates EZH2 protein stability, we measured the abundance of EZH2 in PRMT6-depleted glioblastoma cells and control cells treated with CHX. Immunoblotting analysis showed that the protein half-life of EZH2 was significantly shortened in PRMT6-depleted U87 or LN229 cells (Fig. 3D and Fig. 2A, B), while EZH2 was greatly stabilized in HEK293T cells with abundant PRMT6 expression (Fig. 3E). In 293T cells, *EZH2* plasmid and concentration gradient *PRMT6* plasmid were exogenously transfected to detect whether the expression of EZH2 is affected by PRMT6. The results showed that the expression of EZH2 gradually increases depending on the increase in PRMT6 expression (Fig. 3F). Then, we examined the role of PRMT6 in regulating the protein stability of EZH2 via the proteasome system. In experiments involving PRMT6-depleted glioblastoma cells treated with a proteasome inhibitor (MG132), an increase in EZH2 protein expression was observed (Fig. 3G). Furthermore, we measured the effect of PRMT6 on EZH2 ubiquitination and found that depletion of endogenous *PRMT6* by shRNA increased EZH2 ubiquitination in U87 or LN229 cells (Fig. 3H). These results indicate that PRMT6 enhances the protein stability of EZH2 by inhibiting the ubiquitination degradation of EZH2.

### PRMT6 induces the invasion and migration of glioblastoma cells via EZH2 in vitro

**Fig. 4:**
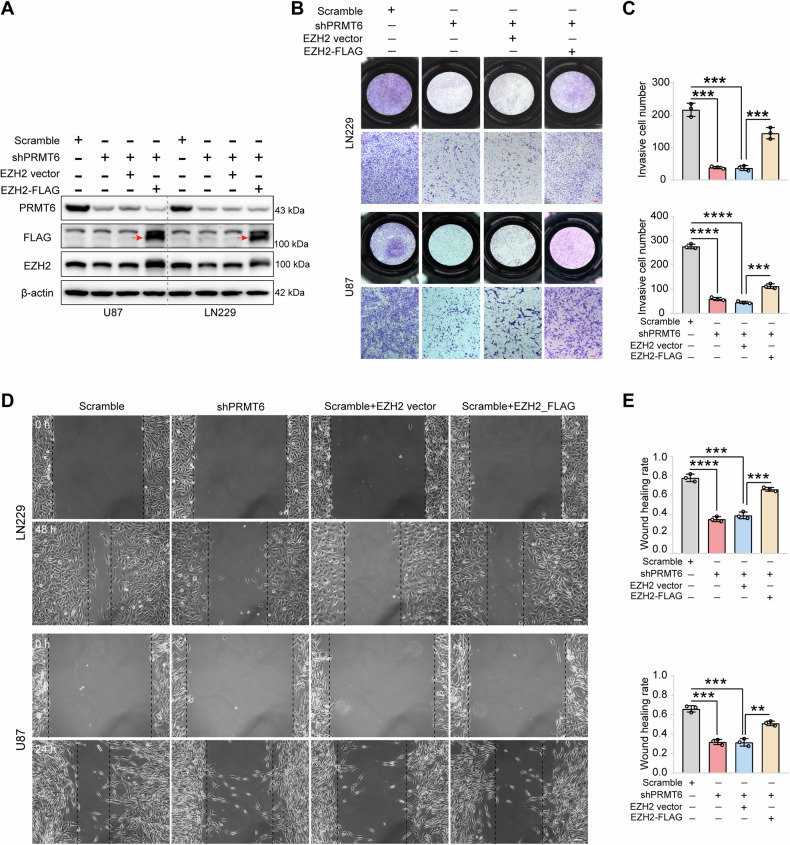
The re-expression of EZH2 negates the inhibition of cell invasion and migration resulting from PRMT6 silencing in glioblastoma cells. **A** Analysis of EZH2 expression in U87 and LN229 cells transfected with Scramble shRNA, shPRMT6, shPRMT6 + EZH2 vector, and shPRMT6 + EZH2-FLAG by immunoblotting. **B** The invasion capacities of U87 and LN229 cells were assessed by the transwell assay following treatment with Scramble shRNA, shPRMT6, shPRMT6 + EZH2 vector, and shPRMT6 + EZH2-FLAG. Bar: 100 μm. **C** Quantification of the invasive cells number. **D** The migration ability of U87 and LN229 cells post-treatment with Scramble shRNA, shPRMT6, shPRMT6 + EZH2 vector, and shPRMT6 + EZH2-FLAG was evaluated by a wound-healing assay. Bar: 200 μm. **E** Quantification of the wound healing rate. **p < 0.01, ***p < 0.001, ****p < 0.0001.

Several studies have shown that highly expressed EZH2 is a key factor in mediating tumor progression [24]. Promoting the transcription of *EZH2* or inhibiting the degradation of EZH2 can aggravate the proliferation and metastasis of tumor cells [34]. In the above studies, we observed that PRMT6 could induce the invasion and migration of glioblastoma cells and enhance the stability of the EZH2 protein by inhibiting ubiquitination degradation. However, whether PRMT6 increases the invasiveness of glioblastoma cells by promoting the expression of EZH2. To investigate the role of EZH2 in PRMT6-mediated glioblastoma cell invasion and migration, we transfected the *EZH2* ORF plasmid into PRMT6-silenced glioblastoma cells to re-expressed EZH2 to construct a rescue cell model. Western blotting results showed that EZH2 protein was re-expressed in PRMT6-deleted LN229 and U87 cells (Fig. 4A). Then, we performed rescue experiments on invasion and migration. Transwell assays revealed that PRMT6 silencing significantly attenuated the invasion of LN229 and U87 cells, while EZH2 overexpression restored the invasive ability of PRMT6-silenced glioblastoma cells (Fig. 4B, C). Wound healing assays also showed that re-expression of EZH2 rescued the inhibition of glioblastoma cell migration ability by PRMT6 silencing (Fig. 4D, E). These rescue experimental results indicate that EZH2 can restore the invasion and migration abilities of glioblastoma cells deprived of PRMT6, that is, PRMT6 induces glioblastoma cell invasion and migration via EZH2.

### PRMT6 is essential in inhibiting the transcription of *TRAF6* in glioblastoma cells

**Fig. 5:**
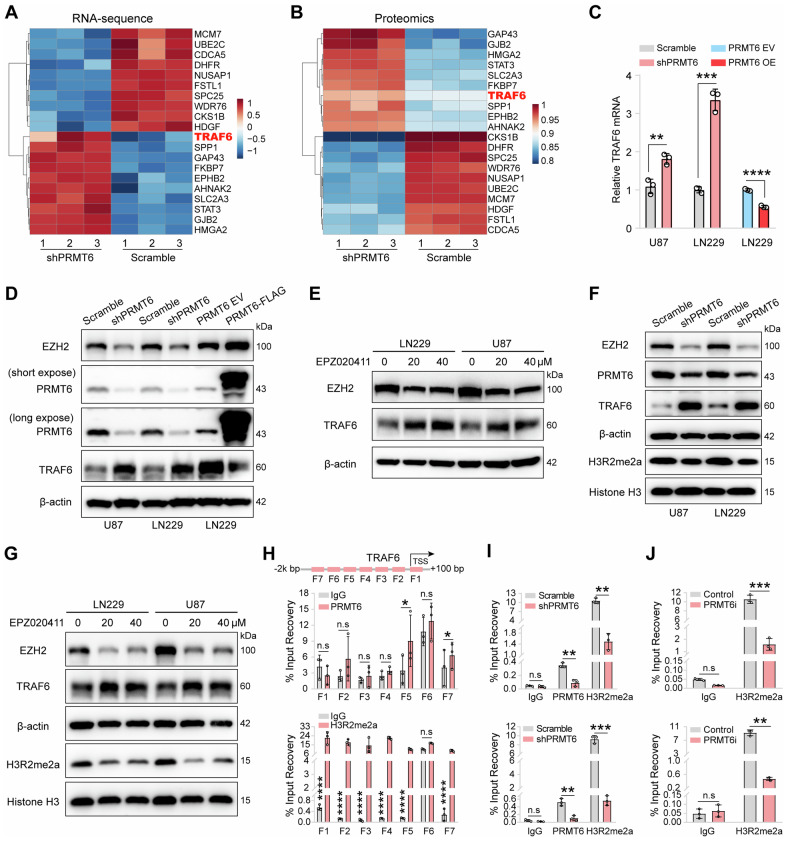
PRMT6 suppresses the transcription of *TRAF6* in glioblastoma cells by H3R2me2a. **A** RNA-seq heatmap illustrating the top 20 most significantly differential genes in PRMT6-depleted U87 cells. **B** Proteomics heatmap displaying the top 20 most significant differential proteins in PRMT6-knockdown U87 cells. **C** qRT-PCR analysis was used to examine the mRNA levels of *TRAF6* in U87 and LN229 cells, both with and without PRMT6 knockdown, as well as in LN229 cells with or without PRMT6 overexpression. **D** The protein levels of TRAF6 were detected in U87 and LN229 cells, both with and without PRMT6 knockdown, as well as in LN229 cells with or without PRMT6 overexpression by western blotting. **E** Immunoblotting analysis was conducted to test the protein expression levels of TRAF6 in U87 and LN229 cells following exposure to the designated concentration of EPZ020411 for 48 h. **F** Protein expression of H3R2me2a was measured via immunoblotting analysis in U87 and LN229 cells, comparing those with or without depletion of PRMT6. **G** The protein expression of H3R2me2a was detected in U87 and LN229 cells treated with specific concentrations of EPZ020411 for 48 h by immunoblotting analysis. **H** The diagrams depict the possible binding sites of PRMT6 or H3R2me2a on the *TRAF6* promoter. ChIP-qPCR testing was performed to identify PRMT6 and H3R2me2a within the *TRAF6* promoter region (+100 to −2000 bp), with IgG serving as a control for comparison. **I**, **J** The ChIP-qPCR experiment was conducted on U87 cells either with or without knockdown of PRMT6 (shPRMT6) (**I**), as well as on U87 cells with or without inhibition of PRMT6 (EPZ020411, PRMT6i) (**J**), to analyze the enrichment levels of PRMT6 and H3R2me2a at the *TRAF6* promoter regions (F5 (upper) and F7 (below)). n.s: no significant, *p < 0.05, **p < 0.01, ***p < 0.001, ****p < 0.0001.

To elucidate the mechanism by which PRMT6 regulates the protein stability of EZH2, we analyzed transcriptomic and proteomic data in shPRMT6 U87 cells and the control cells. As shown in Fig. 5A, B, the heatmaps display the top 20 molecules exhibiting the most significant differences in both RNA and protein levels in U87 cells following PRMT6 knockdown. Among them, the E3 ubiquitin ligase TRAF6 attracted our attention. It is reported that TRAF6 can mediate the ubiquitination of multiple substrate proteins [35]. qRT-PCR and western blotting analysis were conducted to investigate the impact of PRMT6 on TRAF6 expression. The results showed that depletion of PRMT6 increased both the mRNA and protein levels of TRAF6, while overexpression of PRMT6 suppressed the transcription of TRAF6 in glioblastoma cells (Fig. 5C, D). Furthermore, we used EPZ020411 to treat glioblastoma cells for 48 h and obtained similar results, indicating that inhibition of PRMT6 can promote the expression of TRAF6 (Fig. 5E). PRMT6 was confirmed to inhibit gene transcription by modulating histone methylation, particularly asymmetric di-methylation on arginine 2 of histone 3 (H3R2me2a) [18]. Western blotting analysis revealed a global decrease in H3R2me2a and a notable increase in TRAF6 upon PRMT6 knockdown or inhibition (Fig. 5F, G). Further investigation into the 100 bp∼−2000 bp region encompassing the *TRAF6* promoter in glioblastoma cells demonstrated that PRMT6 and H3R2me2a were significantly enriched at 1400 bp∼1101 bp (F5) and 2000 bp∼1701 bp (F7) upstream of the *TRAF6* transcription start site (Figs. 5H and S3A, B). Independent ChIP-qPCR assays confirmed that the occupancy of PRMT6 and H3R2me2a at the *TRAF6* promoter (F5 and F7) loci was decreased in U87 and LN229 cells by PRMT6 silencing or inhibition (EPZ020411) (Figs. 5I, J and S3C). Together, these results uncover an essential role of PRMT6 in regulating TRAF6 expression by adding an activating histone methylation mark (H3R2me2a), that is, PRMT6 suppresses the transcription of *TRAF6* in glioblastoma cells.

### The PRMT6-TRAF6 axis maintains the proteostasis of EZH2

**Fig. 6:**
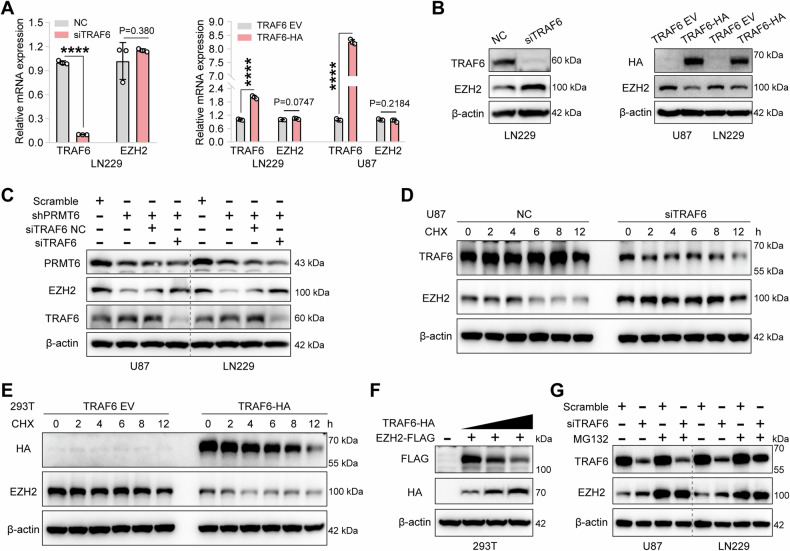
The PRMT6–TRAF6 axis maintains the proteostasis of EZH2. **A** The mRNA levels of *EZH2* were detected via qRT-PCR analysis in LN229 cells that were transfected with *TRAF6* siRNA or NC siRNA, as well as in LN229 and U87 cells that were transfected with *TRAF6* overexpression or vector plasmids. **B** Immunoblot analysis was performed to evaluate TRAF6 and EZH2 protein expression in LN229 cells transfected with *TRAF6* siRNA or NC siRNA, and in LN229 and U87 cells transfected with *TRAF6* overexpression or vector plasmids. **C** Immunoblotting analysis of the protein expression of TRAF6 and EZH2 in PRMT6 knockdown U87 and LN229 cells treated with *TRAF6* siRNA or NC siRNA. **D** Immunoblotting analysis was conducted to investigate the half-life of EZH2 protein in TRAF6 knockdown U87 cells and the control cells. **E** Immunoblotting analysis of HEK293T cells with or without TRAF6 overexpression to determine the protein half-life of EZH2. **F** Protein expression of TRAF6 (HA) and EZH2 (FLAG) was detected in HEK293T cells that were transfected with *EZH2-*FLAG and varying doses of *TRAF6-*HA plasmids by western blotting. **G** U87 and LN229 cells, with or without TRAF6 depletion, were subjected to MG132 (20 μM) treatment for 6 h. The abundance of EZH2 was then assessed by immunoblotting analysis. ****p < 0.0001.

TRAF6 acts as a ubiquitin ligase (E3), which is responsible for the final step of the ubiquitination process by attaching ubiquitin molecules to target proteins [35]. This regulatory function allows TRAF6 to affect the stability and function of various proteins, such as ULK1 and CTLA-4, influencing cellular pathology [36, 37]. Upregulation of TRAF6 expression and decreased EZH2 protein abundance in PRMT6-deficient cells led to the hypothesis that TRAF6 regulates EZH2 protein homeostasis. First, qRT-PCR results showed that compared with control cells, there was no significant difference in *EZH2* transcript levels after silencing or overexpressing TRAF6 in glioblastoma cells (Fig. 6A). Then, western blotting revealed that inhibition of TRAF6 expression in glioblastoma cells promoted the upregulation of EZH2 protein levels, while increased expression of TRAF6 had the opposite effect (Fig. 6B). We transfected the small interfering RNA targeting *TRAF6* into PRMT6-silenced glioblastoma cells to achieve re-inhibition of TRAF6, and subsequently observed an upregulation of EZH2 expression (Fig. 6C). These findings suggest that TRAF6 may be involved in the post-translational regulation of EZH2 protein in glioblastoma cells and that the enhancement of EZH2 protein expression by PRMT6 is mediated by TRAF6. To further assess the effect of TRAF6 on EZH2 protein stability, we examined EZH2 abundance in TRAF6-depleted glioblastoma cells or TRAF6-overexpressed HEK293T cells treated with CHX. The data demonstrated that EZH2 was significantly stabilized in U87 or LN229 cells with TRAF6 absence (Figs. 6D and S4A, C, D), while its half-life was notably shortened in HEK293T cells with TRAF6 overexpression (Figs. 6E and S4B). Moreover, a negative correlation between EZH2 and TRAF6 expression was also observed in HEK293T cells (Fig. 6F). The protein expression of EZH2 in MG132-treated TRAF6-silenced glioblastoma cells was investigated, and western blotting results showed that EZH2 protein levels were further up-regulated compared with TRAF6-silenced glioblastoma cells (Fig. 6G). These results collectively suggest that PRMT6 maintains TRAF6-mediated EZH2 proteostasis in glioblastoma cells.

### TRAF6 interacts with and destabilizes EZH2

**Fig. 7:**
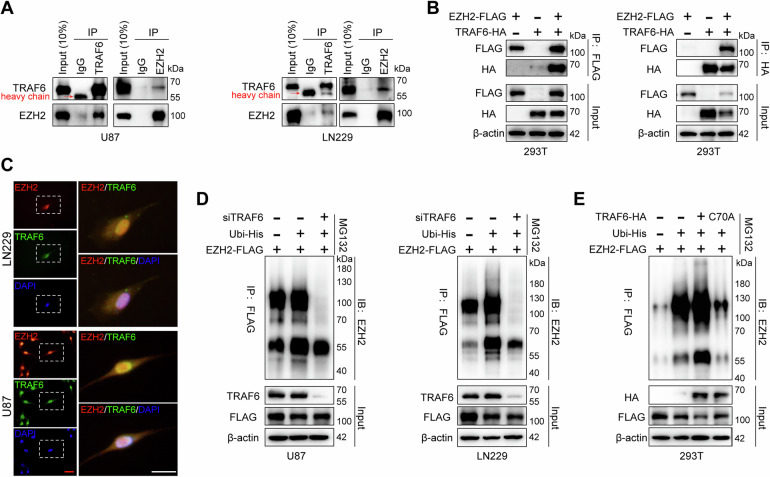
TRAF6 interacts with and destabilizes EZH2. **A** The Co-IP assay was used to assess the interaction between endogenous TRAF6 and EZH2 in U87 and LN229 cells. **B** The Co-IP assay evaluated the interaction between exogenous TRAF6 and EZH2 in HEK293T cells. **C** Under a fluorescence microscope, the expression of EZH2 (Red), TRAF6 (Green), and DAPI (Blue) was observed in LN229 and U87 cells. Bar: 50 μm (Red), 50 μm (White). **D** U87 and LN229 cells were transfected with His*-Ubi* and *EZH2-*FLAG plasmids, with or without TRAF6 depletion, followed by treatment with MG132 (20 μM) for 6 h. The cell lysates underwent immunoprecipitation using an anti-FLAG antibody, and EZH2 ubiquitination was identified by immunoblotting. **E** *TRAF6-*HA WT or *TRAF6-*HA-C70A, *EZH2-*FLAG, and His*-Ubi* plasmids were used to transfect HEK293T cells. Subsequently, the cells were exposed to MG132 (20 μM) for 6 h. Immunoprecipitation of cell lysates was performed using an anti-FLAG antibody, and EZH2 ubiquitination was assessed by western blotting.

To determine the role of TRAF6 in regulating EZH2 protein homeostasis in glioblastoma cells, we performed Co-IP experiments in U87, LN229, and HEK293T cells. Both endogenous and exogenous Co-IP results confirmed that TRAF6 interacts with EZH2 (Fig. 7A, B). Furthermore, cell immunofluorescence images showed that endogenous EZH2 and TRAF6 mainly co-localized in the nuclei of U87 and LN229 cells (Fig. 7C). Then, we investigated whether TRAF6 directly facilitated EZH2 ubiquitination, and we observed a significant decrease in EZH2 ubiquitination in U87 and LN229 cells by ubiquitination assays involving co-transfection of *EZH2*-FLAG and *Ubiquitin*-His plasmids, and *TRAF6* siRNA (Fig. 7D). Conversely, TRAF6 overexpression in HEK293T cells notably increased EZH2 ubiquitination. Moreover, transfection of the E3 ligase-inactivated mutant C70A of TRAF6 in HEK293T cells could decrease EZH2 ubiquitination (Fig. 7E). These findings confirm the interaction and ubiquitination of EZH2 by TRAF6, highlighting TRAF6’s significant role as a mediator in PRMT6-mediated EZH2 upregulation.

### PRMT6 contributes to glioblastoma invasion in vivo

**Fig. 8:**
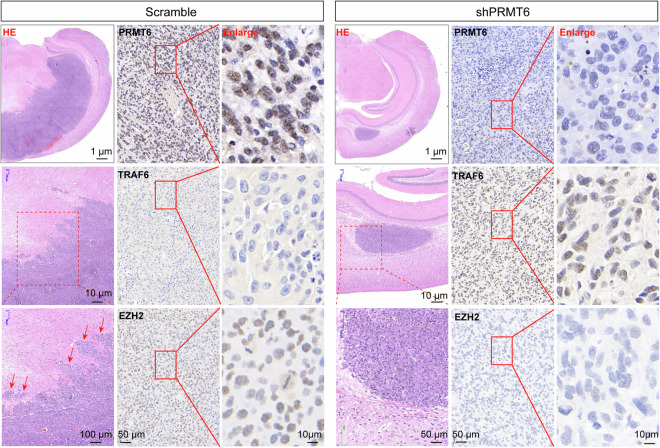
PRMT6 contributes to glioblastoma cell invasion in vivo. U87 cells infected with shPRMT6 and the control cells were injected intracranially into nude mice and the resulting xenograft tumors were analyzed by HE and IHC imaging.

To verify the role of PRMT6 in promoting glioblastoma cell invasiveness in vivo, xenograft experiments were performed in nude mice using PRMT6-silenced U87 cells and control cells. The brain tissues were harvested after mice were sacrificed for sectioning and HE staining of the tumor site. Compared with control tumors, we observed fewer microtumor protrusions, clearer tumor borders, and a slower invasive growth trend in PRMT6-silenced brain tumors (Fig. 8). Moreover, in our previous studies, it has been confirmed that PRMT6 silencing in glioblastoma cells significantly inhibits the growth of transplanted tumors and significantly prolongs the survival time of xenograft mice [19]. Therefore, our study demonstrates that PRMT6 could contribute to the proliferation and invasiveness of glioblastoma cells in vivo, which is the main reason why *PRMT6* is identified as an oncogene in glioma. In addition, IHC staining was used to measure the expression of relevant molecules in the brains of xenograft mice. IHC images showed lower positivity for PRMT6 and EZH2 and higher expression of TRAF6 in PRMT6-silenced tumors compared with control tumors (Fig. 8). These findings from xenograft experiments provide additional evidence supporting the hypothesis that PRMT6 contributes to glioblastoma invasiveness by regulating EZH2 expression via TRAF6.

## Discussion

**Fig. 9:**
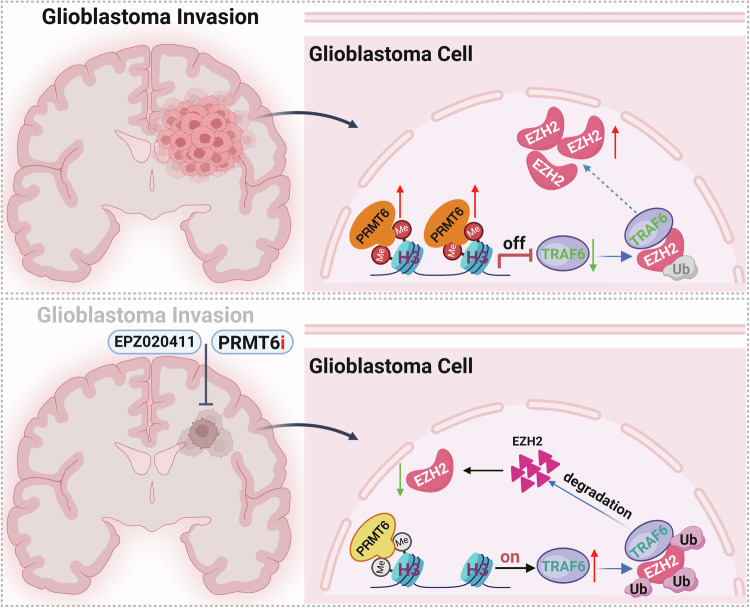
Schematic diagram of glioblastoma cell invasive growth mediated by the PRMT6–TRAF6–EZH2 axis. A model depicting the detailed molecular mechanism underlying the critical role of PRMT6-TRAF6-EZH2 signaling axis in regulation of glioblastoma cell invasive growth.

In the current study, we found that the overexpression of PRMT6 suppressed *TRAF6* transcript levels by facilitating asymmetric dimethylation of histone H3 at arginine 2 (H3R2me2a), which hindered the TRAF6-mediated ubiquitination degradation of EZH2, consequently enhancing the invasion capabilities of glioblastoma cells. (Fig. 9). Our findings not only shed light on the mechanistic aspects of this process but also have clinical implications, highlighting the critical role of the PRMT6–TRAF6–EZH2 axis in the aggressiveness of glioblastoma. Targeting this axis could prove to be a promising therapeutic strategy against glioblastoma.

Invasion is a key hallmark of cancer and a major factor contributing to poor prognosis in glioblastoma patients, with limited effective treatment options available. Our study demonstrates that *PRMT6* acts as an oncogene in glioblastoma, and inhibiting PRMT6 through knockdown or using the inhibitor EPZ020411 notably decreased the invasion and migration of glioblastoma cells. Furthermore, transcriptional expression profile analysis showed that reducing *PRMT6* levels in glioblastoma cells resulted in lower expression of molecules associated with tumor invasion. In other tumors, downregulating PRMT6 has been shown to decrease the aggressive characteristics of endometrial, prostate, and lung cancer cells, leading to a significant reduction in their ability to migrate and invade surrounding tissues [38–40]. Conversely, upregulation of PRMT6 in gastric cancer cells has been found to enhance invasion by inhibiting the transcription of the oncogene protocadherin 7 (PCDH7) through increased levels of H3R2me2as [41]. These findings provide compelling evidence that PRMT6 plays a critical role in promoting tumor invasion. While *PRMT6* has been demonstrated to act as an oncogene in glioblastoma, influencing glioblastoma stem cell division and glioblastoma cell proliferation [19, 20], its precise regulatory mechanisms in glioblastoma invasion are still not well understood.

EZH2, a driver of invasiveness in multiple cancer types, upregulates KRT14 through the H3K27me3 mechanism to enhance peritoneal metastasis in triple-negative breast cancer (TNBC), suggesting that targeted inhibition of EZH2 could potentially impede TNBC metastasis [42]. Studies have demonstrated that elevated levels of EZH2 in gallbladder cancer cells promote tumor cell invasion [43], while inhibition of EZH2 has been shown to suppress migration and invasion in pancreatic cancer [44]. Consequently, EZH2 has emerged as a prominent target for tumor treatment, with several EZH2 inhibitors already being applied in clinical practice [23]. For instance, Tazemetostat (TAZVERIK, Epizyme, Inc.) has been FDA-approved for treating metastatic or locally advanced epithelioid sarcoma in adult and pediatric patients aged 16 and above [45]. However, EZH2 inhibitors have only shown efficacy in certain types of hematologic malignancies, and the clinical advantages of EZH2 inhibitors are still considered inadequate [46]. Therefore, we are dedicated to further investigating the upstream regulatory pathways of EZH2 to address the limitations of current EZH2 inhibitors and provide more effective treatment options for cancer. Li et al.‘s study found that PRMT1 can enhance the stability of EZH2 by methylating EZH2 at R342 [28]. CARM1 facilitates the silencing of EZH2/BAF155 target tumor suppressor genes by methylating BAF155, resulting in the displacement of BAF155 by EZH2 [29]. Furthermore, PRMT5 functionally interacts with EZH2 to suppress CDKN2B expression through epigenetic mechanisms, promoting colorectal cancer (CRC) progression [30]. Our findings demonstrate that PRMT6 is capable of facilitating the post-translational modification of EZH2 in glioblastoma cells. Additional evidence supports that PRMT6 expression enhances the stability of EZH2 protein and inhibits its degradation by the ubiquitin–proteasome system.

Currently, multiple studies have demonstrated that EZH2 protein stability is modulated by a range of post-translational modifications (PTMs), such as ubiquitination, phosphorylation, and acetylation [33]. It is worth noting that the ubiquitin–proteasome system serves as the main pathway for EZH2 degradation. Since the discovery of Praja ring finger protein 1 (Praja1) in 2011, an E3 ubiquitin ligase that directly ubiquitinates EZH2 to reduce its protein levels and inhibit breast cancer progression, several other E3 ubiquitin ligases have been identified to regulate EZH2 protein stability in tumor cells [47, 48]. Particularly, TRAF6, initially recognized as a cytoplasmic adapter protein, has recently been established as a key regulator of EZH2 stability in breast and prostate cancers [28, 49]. Our results demonstrate that TRAF6 interacts with EZH2, facilitating its ubiquitination and subsequent degradation in glioblastoma cells, in line with existing research. Additionally, our further investigations revealed a notable up-regulation in the transcription of *TRAF6* in PRMT6-depleted glioblastoma cells. Arginine methylation modification can impact the biological activity of substrate proteins by methylating them, as well as influence the expression of target genes by methylating histones [18]. Previous studies have demonstrated that TRAF6 can directly regulate its enzymatic activity through arginine methylation [50, 51]. Yet, it remains uncertain whether arginine methylation also plays a role in regulating the expression of TRAF6. Here, our data confirm that PRMT6 mediates the asymmetric dimethylation of histone H3 at arginine 2 (H3R2me2a) to inhibit the transcription of *TRAF6*, resulting in a significant decrease in the expression levels of TRAF6 in glioblastoma cells with PRMT6 overexpressing. Furthermore, the Co-IP experiment confirmed that there is no interaction between PRMT6 and TRAF6 (Fig. S5), and ChIP-qPCR results showed that PRMT6 inhibits the transcriptional regulation of *TRAF6* through H3R2me2a at the *TRAF6* promoter. The regulatory mark of PRMT6-mediated asymmetric dimethylation of histones is a well-known mechanism that can either activate or repress gene expression [52], and our previous study demonstrated that PRMT6 can enhance the transcription of *CDC20* in glioblastoma cells via H3R2me2a [19]. However, these findings appear to be contradictory to the results presented here. The available evidence suggests that differences in the genomic location of target genes and the putative cross-talk between H3R2me2a and neighboring histone marks (e.g. H3K4me1, H3K4me3, H3K27me3, and H3K27ac) may explain the role of H3R2me2a as a transcriptional repressor or activator at target gene promoters [41, 52, 53]. Notably, the current studies confirm that H3R2me2a mainly mediates transcriptional repression of genes [54, 55]. Throughout our study, we identified that TRAF6 is a regulator of EZH2 stability through ubiquitination in glioblastoma cells. Furthermore, PRMT6 suppresses TRAF6 expression through H3R2me2a, indirectly influencing the ubiquitination of EZH2, a critical factor for EZH2 stability. Additionally, rescue experiments demonstrated that PRMT6 enhances glioblastoma cell invasion and migration by modulating TRAF6-mediated EZH2 expression.

In summary, our research illustrates that PRMT6 acts as an epigenetic regulator, suppressing *TRAF6* transcription through histone arginine methylation (H3R2me2a) to inhibit the ubiquitination and degradation of EZH2, thereby promoting invasion and migration of glioblastoma cells. Importantly, our initial experiments demonstrate that a small molecule inhibitor of PRMT6 (EPZ020411) exhibits promising anti-invasive effects on glioblastoma cells in vitro, suggesting the potential for targeted therapy. The PRMT6–TRAF6–EZH2 axis has been identified as a crucial regulator of glioblastoma cell invasion. Nevertheless, we should also recognize several limitations, among which EZH2 has been revealed to drive the malignant progression of glioblastoma by acting on downstream molecules. For instance, EZH2 affects the downstream molecule NF-κB through methylation, enhancing transcriptional activity and promoting self-renewal of glioma stem-like cells [56]. Researchers have found that EZH2 leads to decreased PTEN expression by mediating H3K27me3, thereby activating the PI3K/AKT signaling [57]. Furthermore, the interaction between NEAT1 and EZH2 triggers trimethylation of H3K27, which activates the WNT/β-catenin pathway, thereby increasing the malignancy of glioblastoma [58]. These findings imply that we further explore the detailed mechanisms by which EZH2 regulates the invasion of glioblastoma. Despite the limitations of the study, our results underscore the potential of the PRMT6–TRAF6 axis as a promising target for therapeutic interventions in glioblastoma.

### Supplementary information

Supplementary Material

## Data Availability

The data supporting the findings of this study are available from the corresponding author upon reasonable request.
