# Supplementary material for: PRMT6 facilitates EZH2 protein stability by inhibiting TRAF6-mediated ubiquitination degradation to promote glioblastoma cell invasion and migration
Source: Cell Death Dis. 2024 Jul 23;15(7):524. doi: 10.1038/s41419-024-06920-2 (PMC11266590; doi:10.1038/s41419-024-06920-2)

**Supplementary materials**

**Supplementary methods**

**Quantitative Real-Time PCR assay (qRT-PCR)**

The TRIZOL reagent (Invitrogen, USA) was used to lyse cells for the isolation of total RNA, which was then transcribed in reverse into cDNA utilizing a cDNA synthesis kit (Invitrogen, USA). Subsequently, qRT-PCR was conducted using SYBR Green (Takara, Beijing, China) in an ABI 7000 thermal cycler. The analysis of RNA expression was performed using the 2^−ΔΔCT^ method, with *β-actin* utilized as an internal control for normalization purposes. The primer sequences were obtained from Sangon Biotechnology (Shanghai, China) and are listed in Supplementary Table S2.

**Western blotting assay**

To extract total protein, cells were lysed with RIPA lysis buffer containing protease inhibitors (Beyotime, Shanghai, China). The total protein concentration was assessed with the BCA kit (Invitrogen, CA, USA). Equivalent amounts of total protein (30 µg) were subjected to electrophoresis on 10% -12% SDS-PAGE and subsequently transferred onto a nitrocellulose filter (NC) membrane (Millipore, MA, USA). Membranes were incubated with primary antibodies overnight at 4°C after blocking with 5% nonfat dry milk in TBST for 1 h at RT. After washing with TBST, the membranes were incubated for 1 h at RT with HRP-labeled secondary antibodies (Jackson, Lancaster, USA). Protein expression signal was measured using an enhanced chemiluminescence system (Millipore, MA, USA). The relative quantity of protein expression was analyzed using ImageJ software. Primary antibodies used for western blotting were: anti-PRMT6 (CST, #14641, 1:1000), anti-EZH2 (Proteintech, #66476-1-lg, 1:3000), anti-TRAF6 (Abcam, ab40675, 1:5000), anti-H3R2me2a (Abcam, ab176845, 1:500), anti-Histone H3 (Abcam, ab1791, 1:2000), anti-FLAG (CST, #14793, 1:3000), anti-HA (CST, #3724, 1:3000), and anti-β-actin (Proteintech, #66009-1-lg, 1:10000).

**Protein half-life assay**

Following treatment with a specific condition, U87, LN229, and HEK293T cells were exposed to 100 µg/ml of cycloheximide (CHX, Selleck, USA). The cell lysates were obtained at 0, 2, 4, 6, 8, and 12 h after treatment, followed by analysis using western blotting.

**Ubiquitylation of EZH2 *in vivo* and *in vitro***

The control, NC, shPRMT6 LN229, and U87 cells were transfected with a His-ubiquitin plasmid for 48 h. Subsequently, the cells were exposed to 20 µM MG132 (MCE, USA) for 6 h. Following this, cell lysis was performed using NP-40 lysis buffer, and the resulting cell lysates were subjected to immunoprecipitation with anti-EZH2 antibody and Protein A/G agarose beads. The beads were then washed with TBS buffer and analyzed using western blotting with anti-EZH2 antibody. HEK293T, U87 and LN229 cells were co-transfected with *EZH2*-FLAG, His-*Ubiquitin*, and *TRAF6*-HA or siTRAF6 for 48 h. Subsequently, the cells were exposed to 20 µM MG132 for 6 h. Immunoprecipitation of EZH2 protein, which contained ubiquitylated EZH2, was performed from the cell lysates using FLAG-beads. The immunoprecipitated complex was extensively washed with TBS buffer and subjected to immunoblotting analysis with anti-EZH2 antibody.

**Immunofluorescence (IF)**

U87 and LN229 cells were cultured on 35 mm sterile coverslips at an appropriate density for 24 h. After a pre-cold PBS wash, the cells were fixed with 4% PFA for 30 min. Subsequently, they were exposed to 0.5% Triton X-100 for 10 min at RT. Following another PBS wash, the cells were blocked with 10% BSA at RT for 1 h before undergoing overnight incubation at 4 °C with the desired primary antibodies. The next day, fluorescent secondary antibodies (Alexa Fluor® 488 or Alexa Fluor® 555) were added to the cells and incubated at RT for 1 h in the dark. Finally, the cell nuclei were stained with DAPI, and images of IF staining were captured by a fluorescence microscope. The primary antibodies used for IF were anti-EZH2 (Proteintech, #66476-1-lg, 1:400) and anti-TRAF6 (Abcam, ab40675, 1:200).

**Immunohistochemistry (IHC)**

Fresh human glioma tissue or mouse brain tissue samples were fixed with 4% PFA and embedded in paraffin. Following this, 5 µm slices were cut using a microtome (Leica, Germany) and then underwent deparaffinization, dehydration, and heat-mediated antigen retrieval. Endogenous catalase was removed with 3% H_2_O_2_‐methanol, and the tissue slices were then incubated with the specified primary antibodies at 4 °C overnight. The next day, the sections were rinsed with PBS and then exposed to biotinylated secondary antibodies at RT for a period of 60 min. The samples were treated with peroxidase solution for a total of 30 min, then stained with DAB reagent and counterstained with hematoxylin. The sections were then examined and analyzed under an optical microscope. ImageJ software was used for the quantification of IHC staining images. The IHC primary antibodies used were: anti-PRMT6 (Proteintech, #15395-1-AP, 1:100), anti-EZH2 (Proteintech, #66476-1-lg, 1:50) and anti-TRAF6 (Santa cruz, sc-8409, 1:50).

**Bioinformatics analysis**

GlioVis website (http://gliovis.bioinfo.cnio.es/) was utilized for the analysis of *PRMT6* expression levels in Proneural (PN), Classical (CL), and Mesenchymal (MES) subtypes, and survival prognosis of patients with MES subtype glioblastoma, with data obtained from the TCGA and CGGA databases. Statistical analysis and graphical representations were performed with GraphPad Prism 8.0 software (La Jolla, CA, USA).

**Supplementary figures**


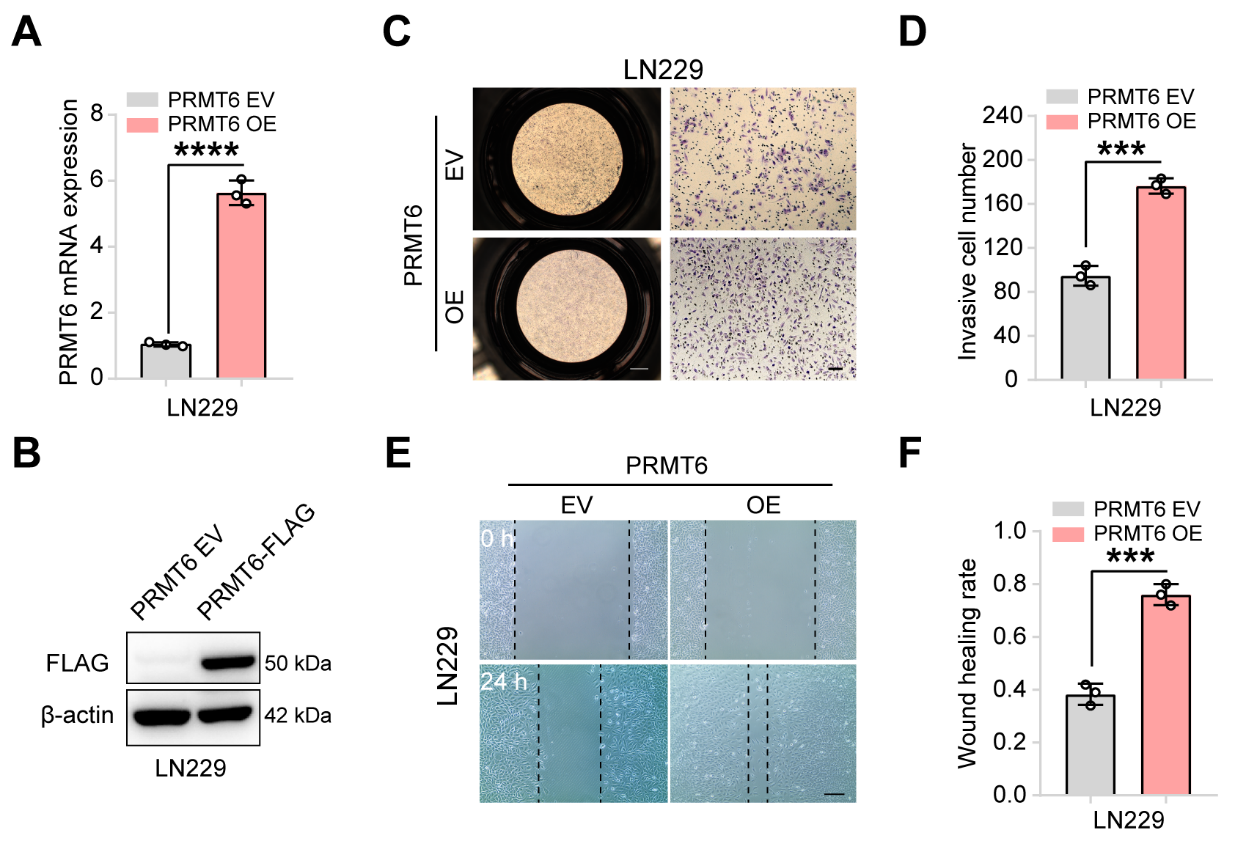


**Fig. S1 PRMT6 promotes the invasion and migration of glioblastoma cells. A** qRT-PCR analysis was used to detect mRNA levels of *PRMT6* in LN229 cells transfected with *PRMT6* overexpression or vector plasmids. **B** The protein levels of PRMT6 was detected in LN229 cells transfected with *PRMT6* overexpression or vector plasmids by immunoblotting. **C** Transwell assay was utilized to assess the invasion capacities in LN229 cells transfected with *PRMT6* overexpression or vector plasmids. Bar: 100 μm. **D** Quantification of the invasive cells number. **E** Wound-healing assay was performed to measure the migration ability of PRMT6-overexpression LN229 cells or control cells. Bar: 100 μm. **F** Quantification of the wound healing rate. ***p < 0.001, ****p < 0.0001.


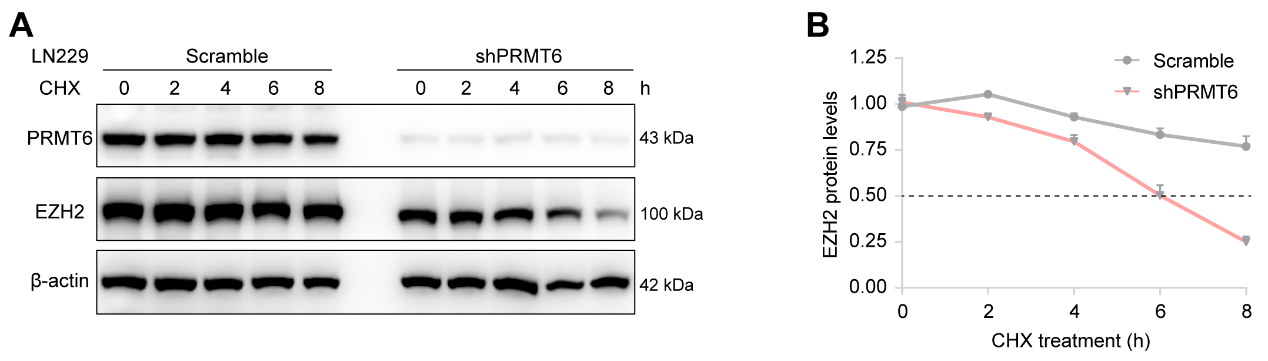


**Fig. S2 PRMT6 improved the protein stability of EZH2 through the ubiquitin-proteasome pathway. A** Immunoblotting analysis of LN229 cells with and without PRMT6 knockdown to measure and quantify the protein half-life of EZH2. **B** Quantifications of EZH2 protein half-life result in LN229 cells.


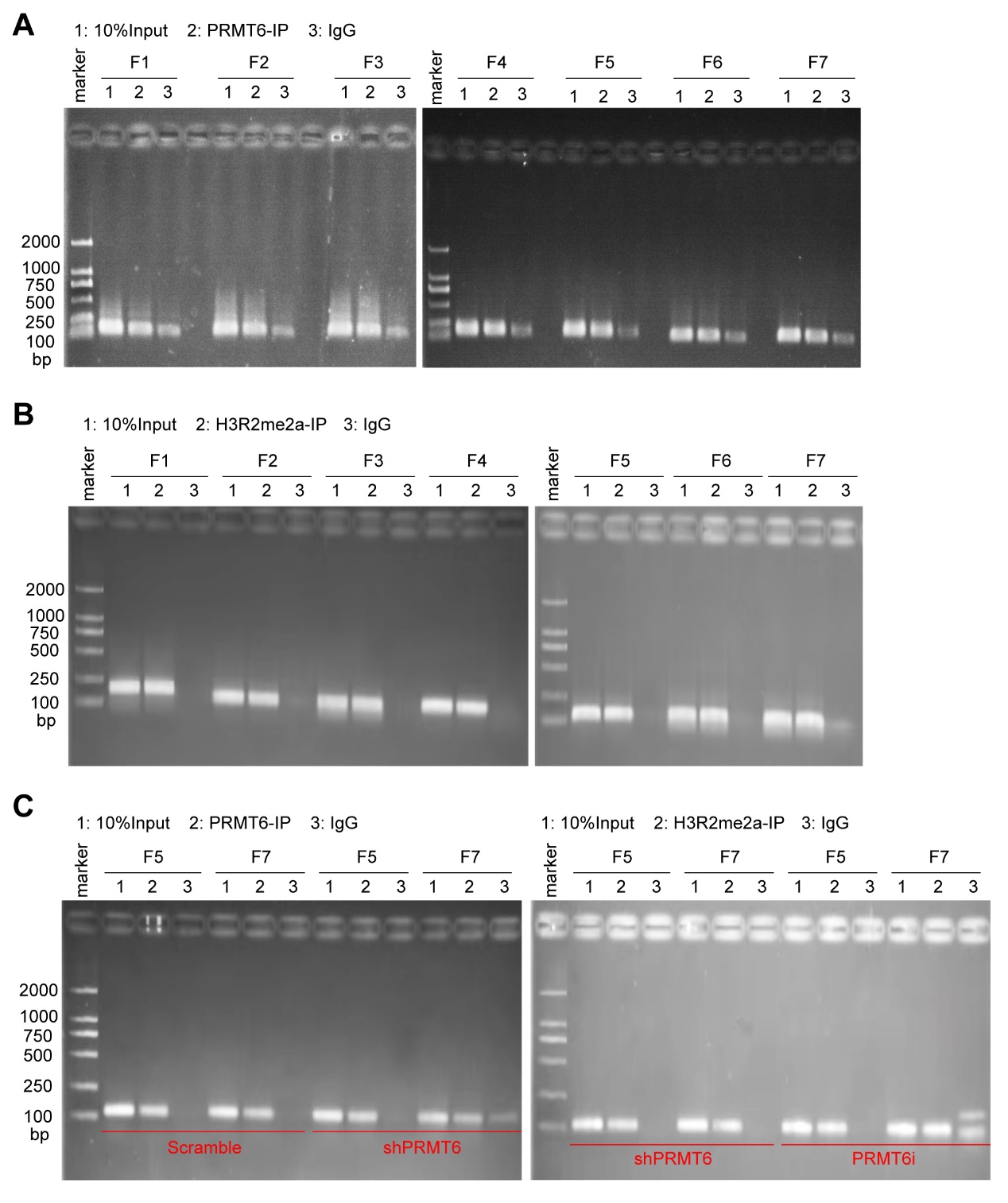


**Fig. S3 PRMT6 inhibited the transcription of *TRAF6* in glioblastoma cells by H3R2me2a. A**, **B** Agarose gel electrophoresis was used to analyze the ChIP-qPCR products of PRMT6 (**A**) and H3R2me2a (**B**) in the promoter region of *TRAF6*. **C** The ChIP-qPCR products of PRMT6 and H3R2me2a in the promoter region of *TRAF6* (F5 and F7) in U87 cells with or without PRMT6 knockdown (shPRMT6) and U87 cells with or without PRMT6 inhibition (EPZ020411, PRMT6i) were analyzed on agarose gel electrophoresis.


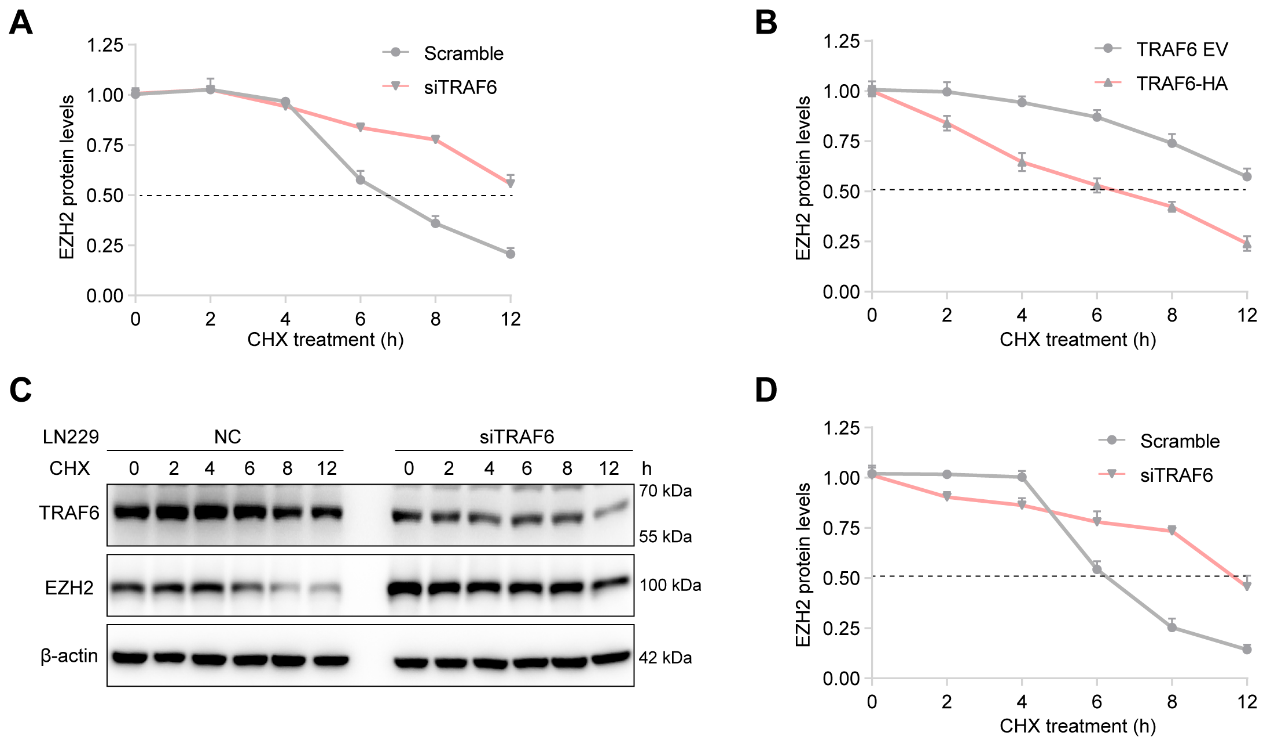


**Fig. S4 The PRMT6-TRAF6 axis maintains the proteostasis of EZH2. A, B** Quantifications of EZH2 protein half-life results in U87 (**A**) and HEK293T (**B**) cells. **C** Immunoblotting analysis of LN229 cells with or without TRAF6 knockdown to examine the protein half-life of EZH2. **D** Quantifications of EZH2 protein half-life result in LN229 cells.


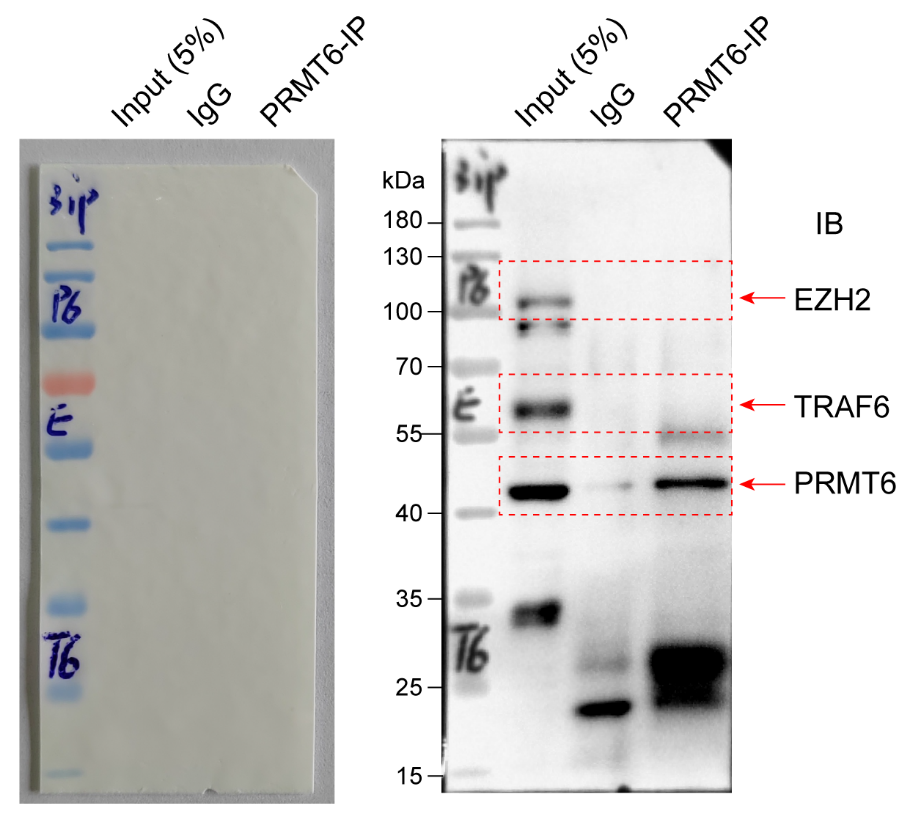


**Fig. S5 PRMT6 does not interact with TRAF6 or EZH2.** The Co-IP assay was used to evaluate the interaction between endogenous PRMT6 and TRAF6 or EZH2 in U87 cells.

**Supplementary tables**

**Table S1.** Sequences of siRNAs and shRNAs.

| **shRNA/siRNA** | **Sequence (5'-3')** |
| --- | --- |
| Scramble | CCGGCAACAAGAT GAAGAGCACAACTCGAGTTGGTGCTCTTCATCTTGTTGTTTTT |
| shPRMT6 | CCGGGCCCAGTTTGAGATGCCTTATCTCGAGATAAGGCATCTCAAACTGGGCTTTTTG |
| siRNA_NC | UUCUCCGAACGUGUCACGUTT |
| siTRAF6 | GTTCATAGTTTGAGCGTTA |

**Table S2.** Primer sequences for RT-qPCR and ChIP-qPCR.

| **Genes** | **Sequence (5'-3')** |
| --- | --- |
| *PRMT6* | F: TGGCTTTGCCATCTGGTTCCAG |
|  | R: TAGAGGAGCGCCTGTTTCCAGT |
| *EZH2* | F: GACCTCTGTCTTACTTGTGGAGC |
|  | R: CGTCAGATGGTGCCAGCAATAG |
| *TRAF6* | F: CAATGCCAGCGTCCCTTCCAAA |
|  | R: CCAAAGGACAGTTCTGGTCATGG |
| *β-actin* | F: TCCTGTGGCATCCACGAAACT |
|  | R: GAAGCATTTGCGGTGGACGAT |
| *TRAF6* F1 | F: GCAACTGCCCTTCATACATCA |
|  | R: GTTCTGGCCTGTGAGCTATGA |
| *TRAF6* F2 | F: GCCTGGTAAGGTAGACATTATCC |
|  | R: TGGAGAAAAGGAAGAGCAAGA |
| *TRAF6* F3 | F: AGAACCTGAGAACCAGTGATA |
|  | R: TAGTATGGGCTCAATAAATGT |
| *TRAF6* F4 | F: CTCAAGCCATCCTCCCACC |
|  | R:AGACCCCATCTTTACACAAAATTTA |
| *TRAF6* F5 | F: TCTGGCTTCAGGATACATACG |
|  | R: GCTGGAGAAGCACAAAAGAGT |
| *TRAF6* F6 | F: GATGAAGGGGAAGAGCAGGTA |
|  | R: GGGCTAGATCGCTTGACCTTC |
| *TRAF6* F7 | F: CGTCGCCATCCTCATTCC |
|  | R: GGCTTTCTTCCTGCTCGG |

**Full and uncropped western blots**


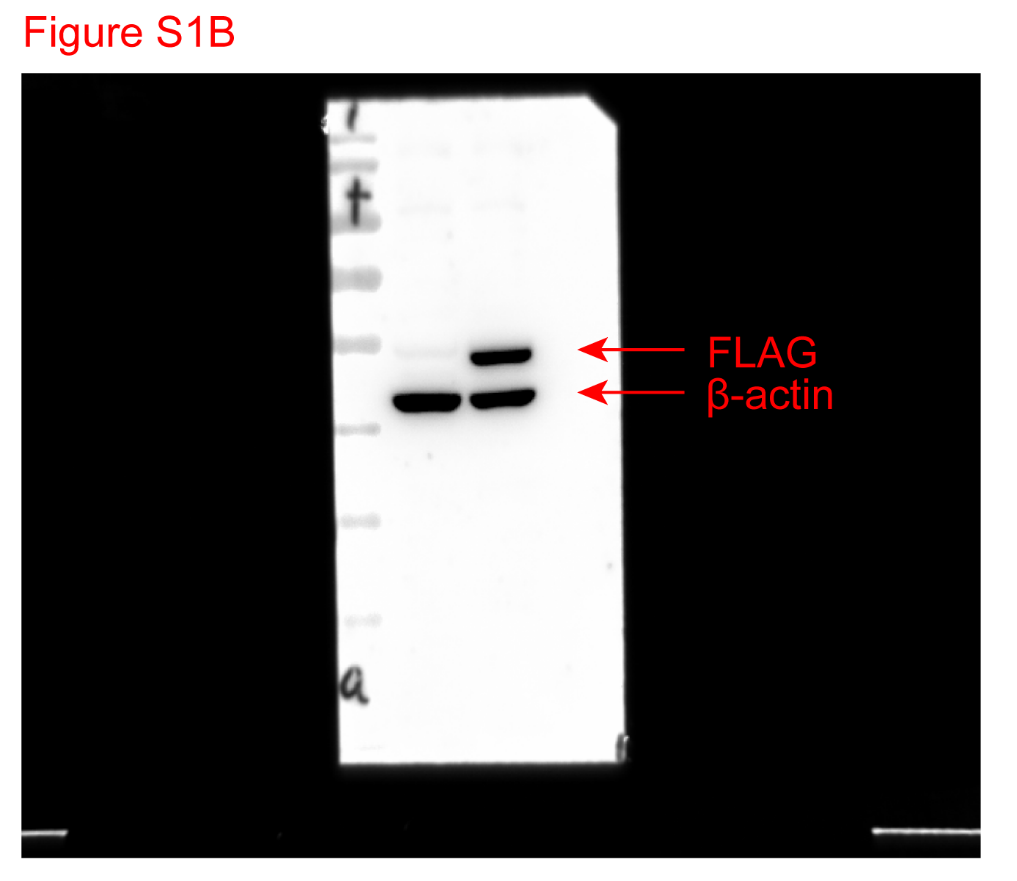


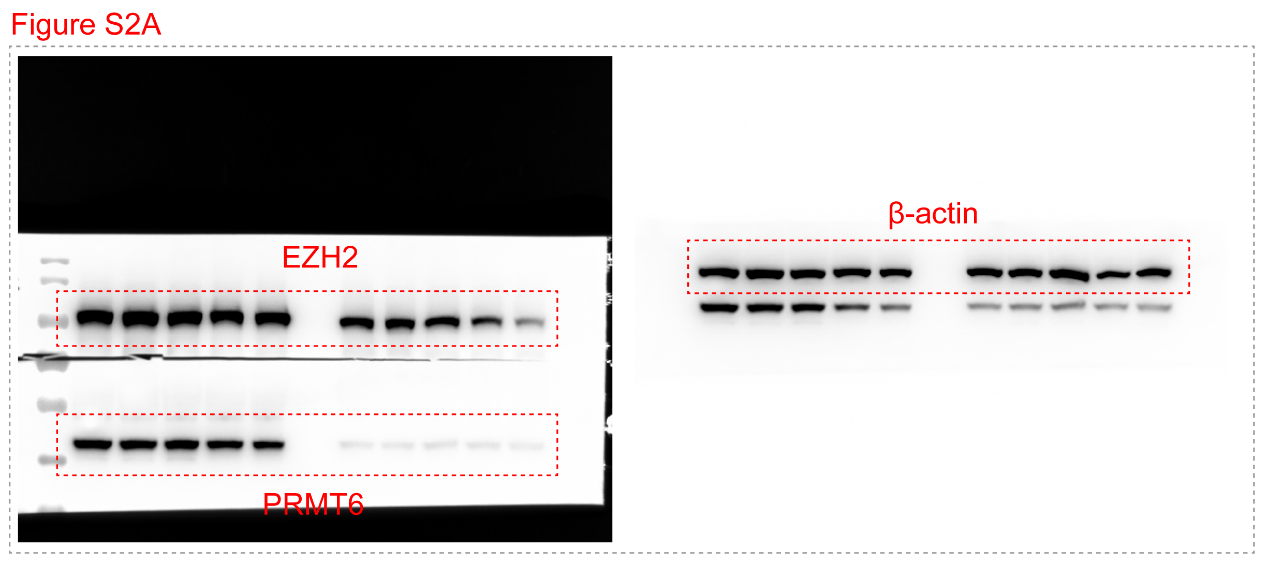


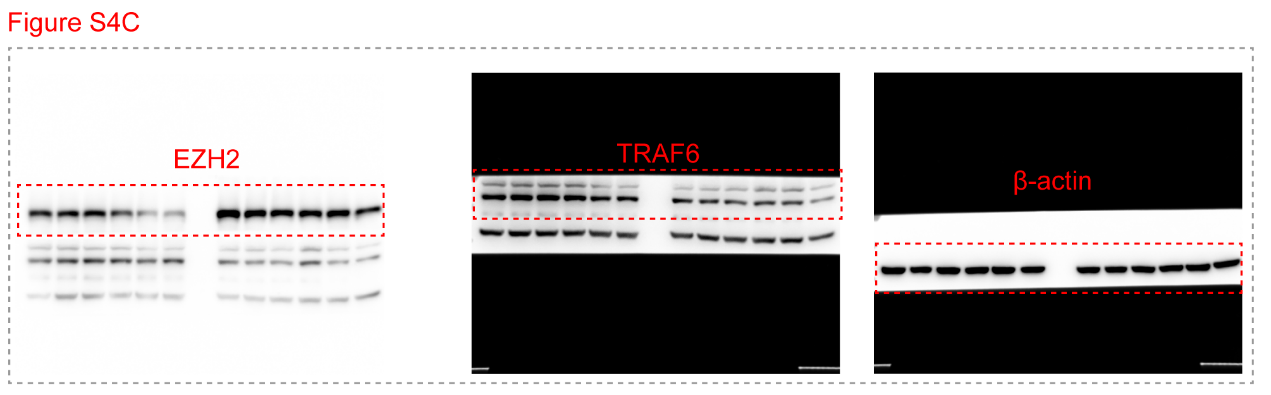


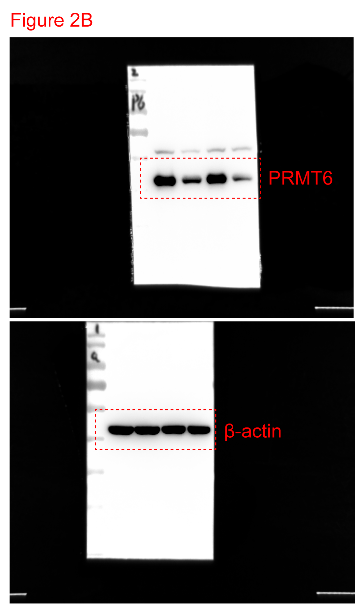


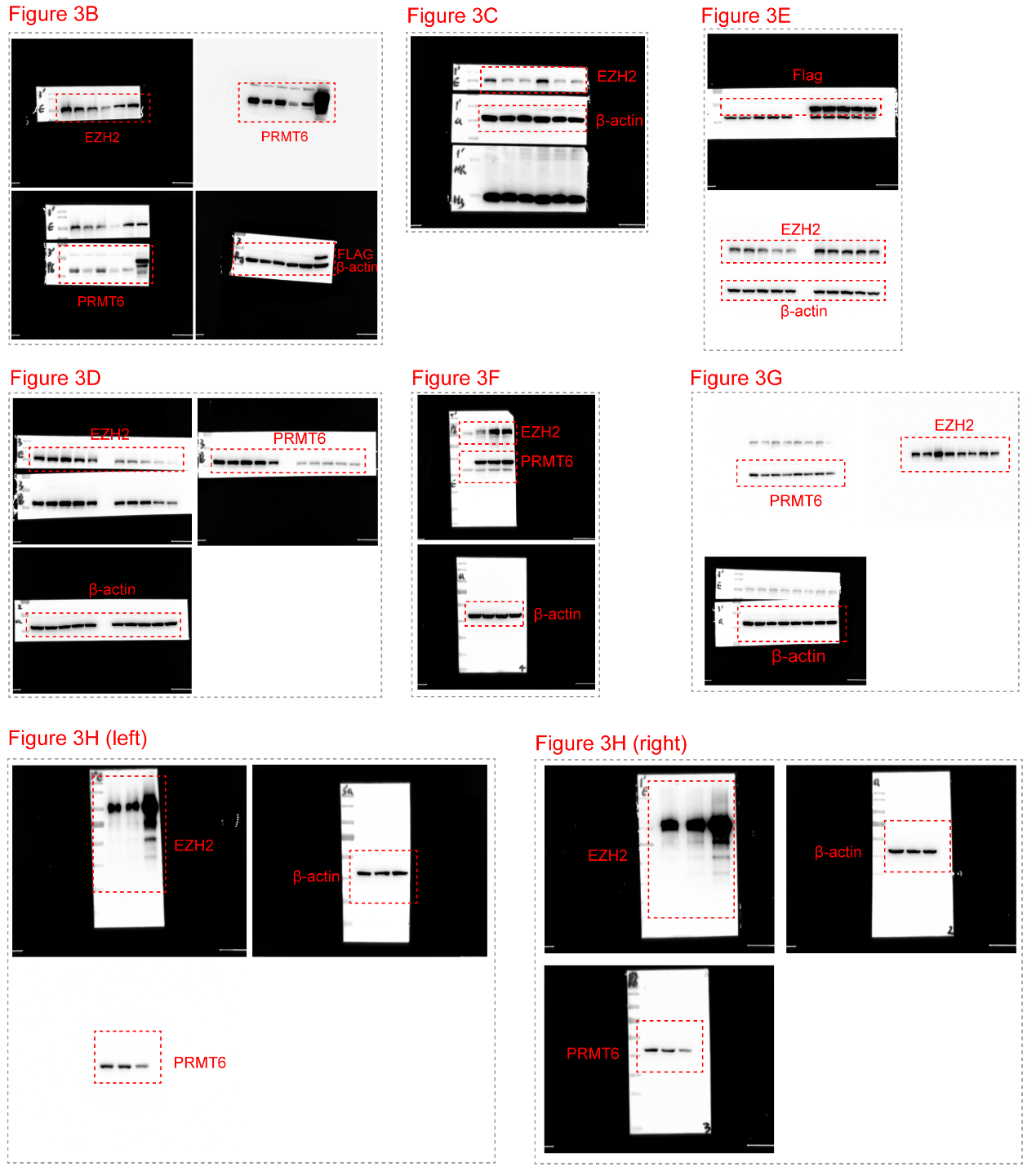


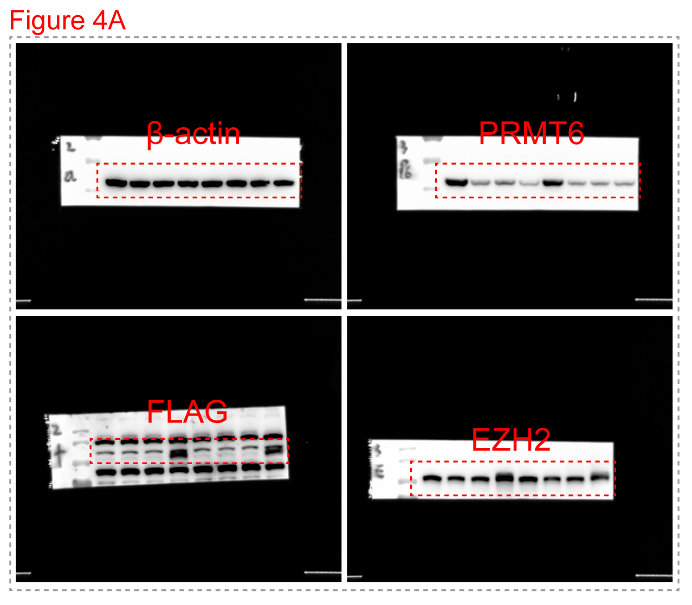


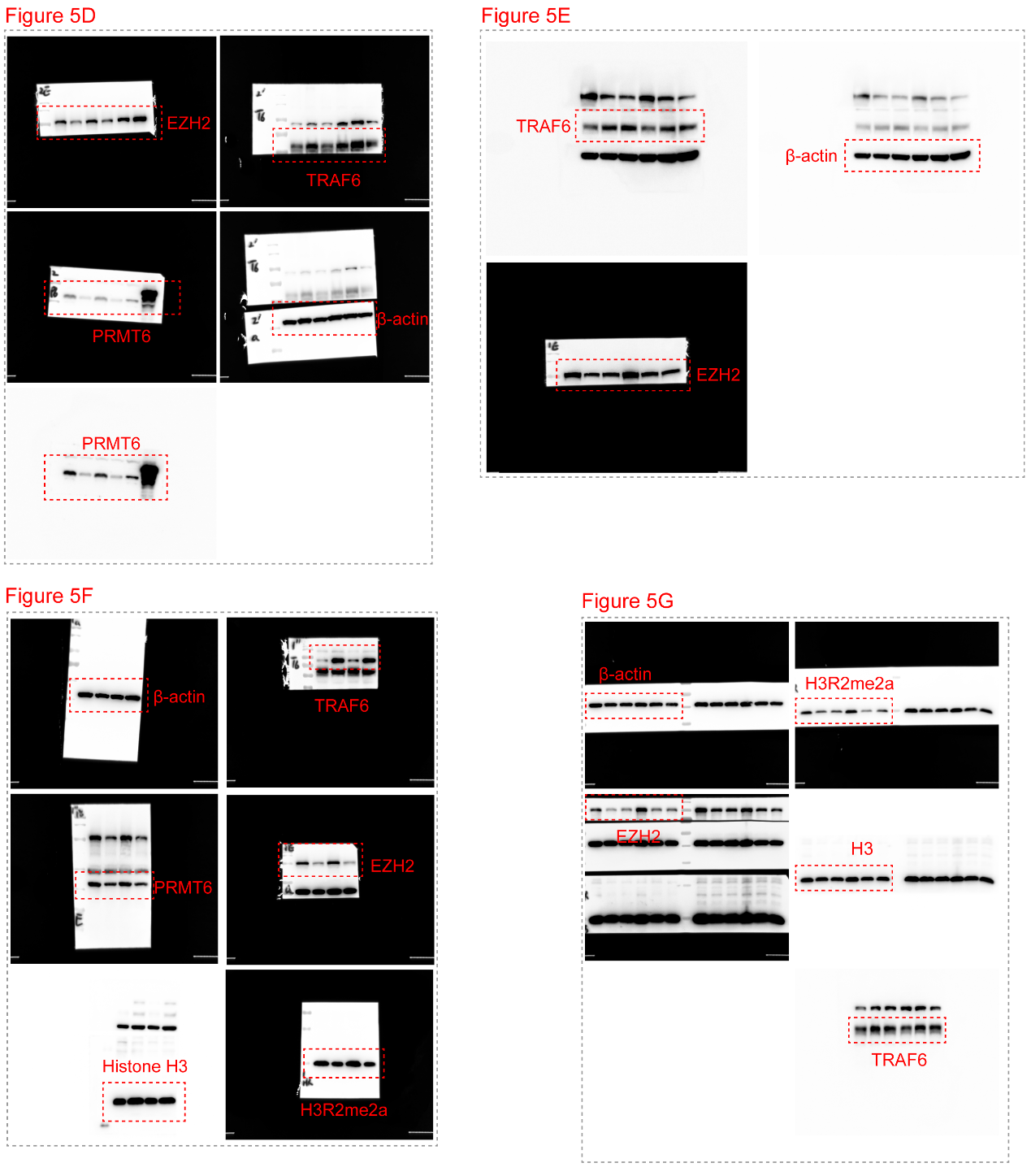


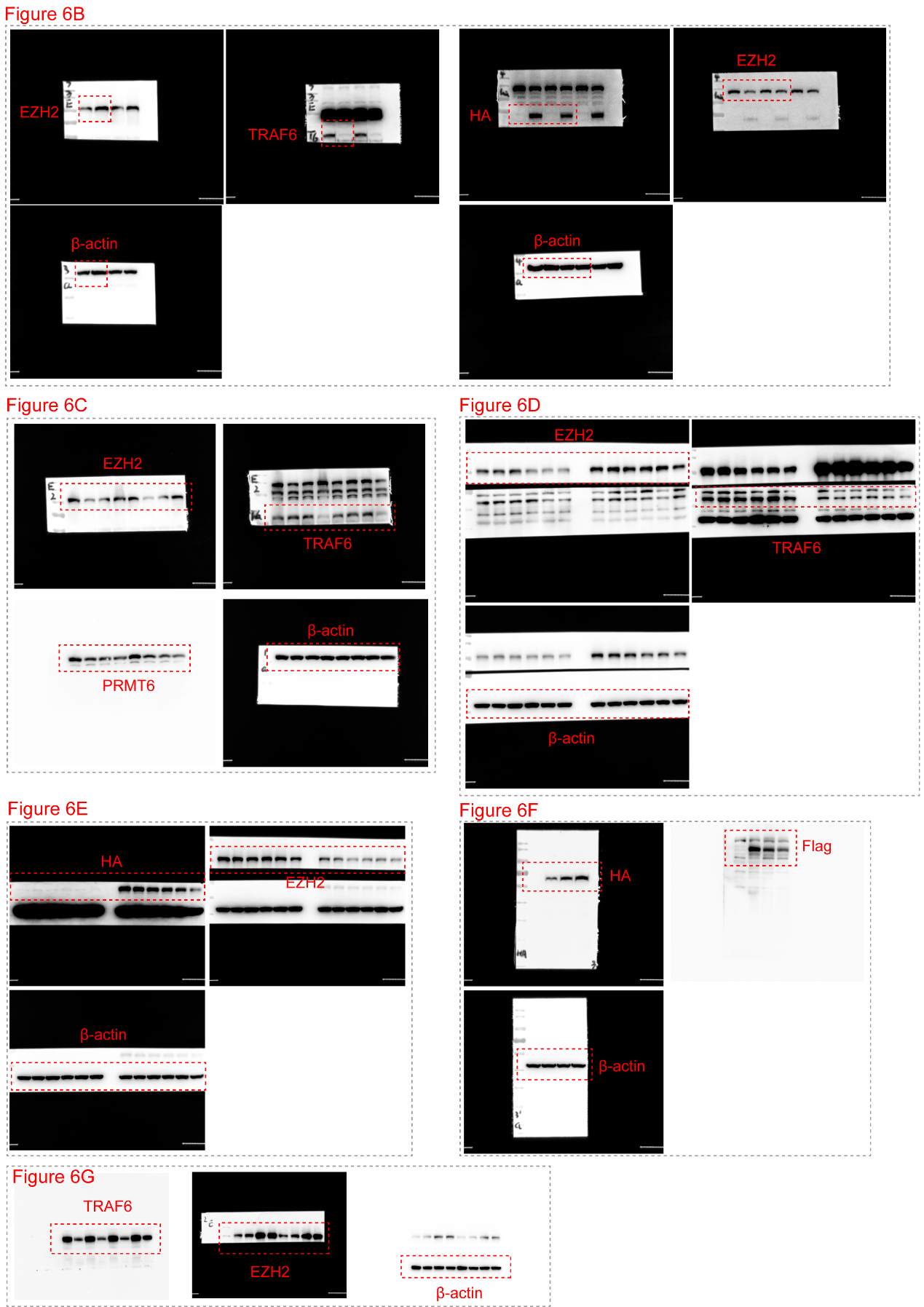


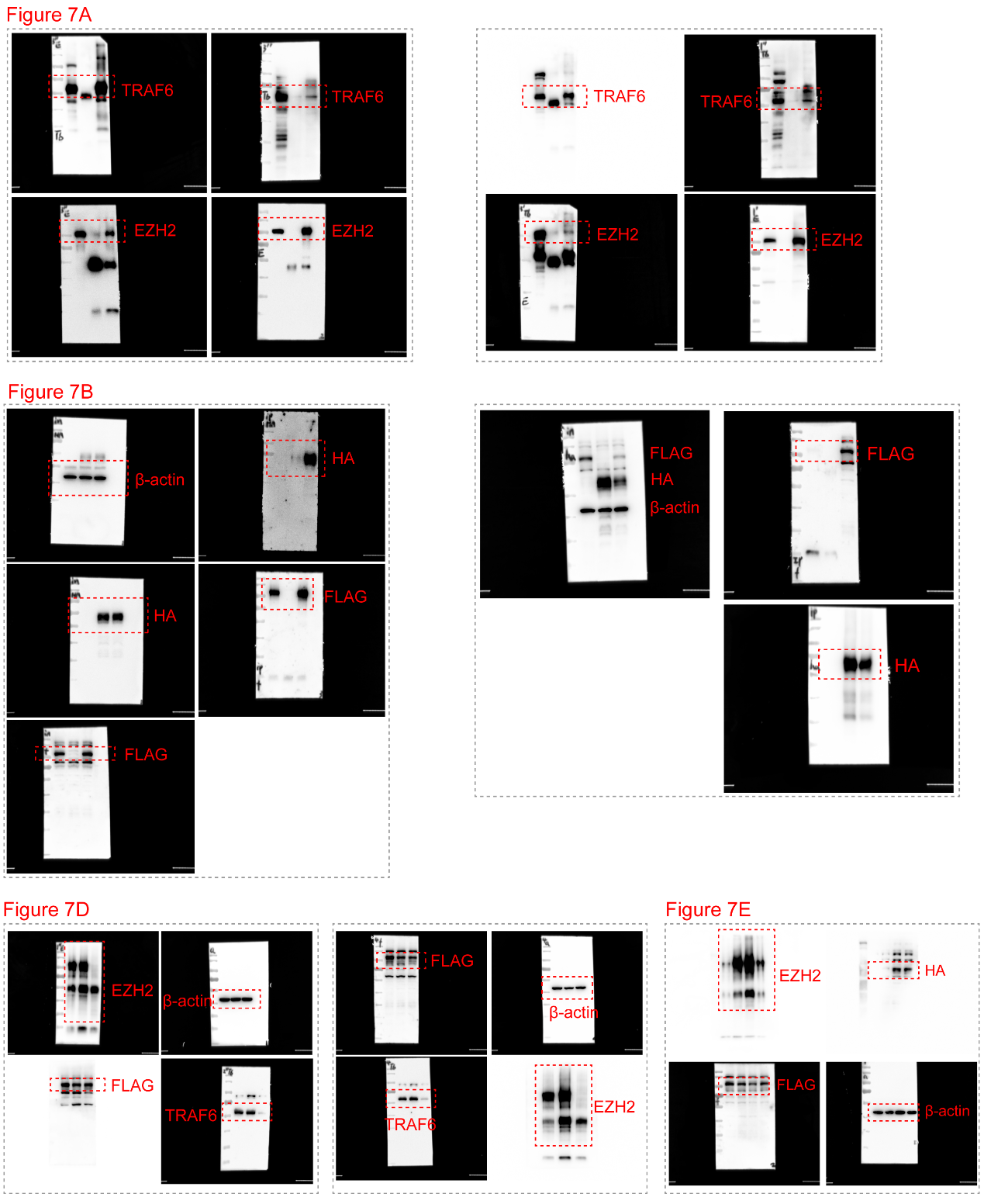

Supplement: Supplementary file 1 — Supplementary Material [file 41419_2024_6920_MOESM1_ESM.docx]
